# Novel miRNA-SSRs for Improving Seed Hardness Trait of Pomegranate (*Punica granatum* L.)

**DOI:** 10.3389/fgene.2022.866504

**Published:** 2022-04-12

**Authors:** Prakash Goudappa Patil, Nripendra Vikram Singh, Abhishek Bohra, Shivani Jamma, Manjunatha N, Venkatesh S. C, Dhinesh Babu Karuppannan, Jyotsana Sharma, Rajiv A. Marathe

**Affiliations:** ^1^ ICAR-National Research Centre on Pomegranate (NRCP), Solapur, India; ^2^ ICAR-Indian Institute of Pulses Research (IIPR), Kanpur, India; ^3^ Dept. of Biotechnology and Crop Improvement, University of Horticultural Sciences (UHS), Bagalkot, India

**Keywords:** functional markers, miRNA, pomegranate, SSR, seed type

## Abstract

Present research discovered novel miRNA-SSRs for seed type trait from 761 potential precursor miRNA sequences of pomegranate. SSR mining and BLASTx of the unique sequences identified 69 non-coding pre-miRNA sequences, which were then searched for BLASTn homology against Dabenzi genome. Sixty three true pri-miRNA contigs encoding 213 pre-miRNAs were predicted. Analysis of the resulting sequences enabled discovery of SSRs within pri-miRNA (227) and pre-miRNA sequences (79). A total of 132 miRNA-SSRs were developed for seed type trait from 63 true pri-miRNAs, of which 46 were specific to pre-miRNAs. Through ePCR, 123 primers were validated and mapped on eight Tunisia chromosomes. Further, 80 SSRs producing specific amplicons were ePCR-confirmed on multiple genomes *i.e.* Dabenzi, Taishanhong, AG2017 and Tunisia, yielding a set of 63 polymorphic SSRs (polymorphism information content ≥0.5). Of these, 32 miRNA-SSRs revealed higher polymorphism level (89.29%) when assayed on six pomegranate genotypes. Furthermore, target prediction and network analysis suggested a possible association of miRNA-SSRs *i.e.* miRNA_SH_SSR69, miRNA_SH_SSR36, miRNA_SH_SSR103, miRNA_SH_SSR35 and miRNA_SH_SSR53 with seed type trait. These miRNA-SSRs would serve as important genomic resource for rapid and targeted improvement of seed type trait of pomegranate.

## Introduction

Pomegranate (*Punica granatum* L.) native to central Asia and is widely cultivated in tropical and subtropical regions. The most of the commercially cultivated pomegranate varieties in India are medium-to hard-seeded types. Therefore, breeding for the soft-seeded varieties is top priority for consumer point of view. Hard seeds of pomegranate are not preferred for consumption because they are too hard to chew and swallow (Xia et al., 2019). However, phytosterols and special fatty acid such as punicic acid found in pomegranate seeds offer a variety of health benefits. There is an urgent need for breeding soft-seeded pomegranate cultivars that provide new products for the market and contribute to enhanced farmer incomes. However, the genetic architecture of seed type trait is largely unknown. Therefore, understanding the genetic mechanisms underlying seed type would facilitate the development of new commercially viable pomegranate varieties (Luo et al., 2018). There are three types of pomegranate varieties: soft-seeded (seed hardness<3.67 kg cm^−2^), semi soft-seeded (seed hardness from 3.67 to 4.2 kg cm^−2^), and hard-seeded (seed hardness>4.2 kg cm^−2^) (Lu, 2006). Research has shown that the differences in the hard-seeded and soft-seeded cultivars could be attributed to the variation in expressions of genes or transcription factors *CCR, CAD, CelSy, SuSy, CCoA-OMT, MYB, WRKY and MYC* involved in lignin and cellulose biosynthesis (Xue et al., 2017). The divergence between hard- and soft-seeded pomegranates is the embodiment of germplasm diversity in pomegranate. Owing to the complexity of metabolic synthesis, underlying the degree of seed hardness of pomegranate is influenced by environmental factors and genetic background (Zhang et al., 2020). The softness of seeds is a desirable economic trait that enhances the consumptive qualities of fruits, but the complete soft-seeded pomegranate is restricted to a narrow ecological region and requires cold protection at low temperatures (Song et al., 2012). The quantitative nature of seed type may likely contain a regulator gene conferring tolerance to adverse environmental conditions (Lu, 2006). Deciphering ecological and evolutionary forces shaping the population structure of the soft-seeded pomegranate will help us understand the genetic makeup of the seed trait (Zhang et al., 2020). Investigations on the formation of the soft seed will elucidate the mechanism of lignin synthesis that contributes to plant growth and development and confers resistance to biotic and abiotic stresses (Liu et al., 2018).

Non-coding (nc) RNAs including miRNAs are known to regulate multiple aspects of plant growth and development and plant’s response to a variety of stresses (Millar, 2020). High throughput sequencing has emerged as a promising tool to discover miRNAs and their gene targets at large scale in different plant species including pomegranate (Singh et al., 2017; Luo et al., 2018). Deep sequencing of small RNA libraries constructed from seeds at 60 and 120 DAF of soft-seeded and hard-seeded pomegranate enabled identification of miRNAs specific to seed type trait (Luo et al., 2018). The study suggested that a complex biological process mediated by miRNA-mRNA networks controls the seed type in pomegranate. The presence of genetic variations in pre-miRNAs and miRNAs are known to affect quantitative trait expression (Ferrao et al., 2015), hence DNA markers may be developed from the miRNA regions to assist the procedure of trait improvement (Kumar et al., 2017). Study by Joy et al. (2018) showed presence of SSRs in pre-miRNAs and the authors proposed a role for alternative splicing in creating mature RNA isoforms in response to stress. Therefore, earlier a large scale development of SSR markers from coding regions of miRNA sequences has been reported. For instance, Chen et al. (2010) performed a comprehensive analysis of the SSR prediction in 8,619 pre-miRNA sequences from 87 species from nine different taxonomic groups.

In pomegranate, recent advances such as whole genome sequencing (Akparov et al., 2017; Qin et al., 2017; Yuan et al., 2018; Luo et al., 2020), and transcriptome profiling (Ophir et al., 2014; Saminathan et al., 2016; Luo et al., 2018) have paved the way for large-scale discovery of functional molecular markers for use in genetic improvement programs (Mondal and Ganie, 2014). A previous research on genome wide discovery of miRNA-SSRs in pomegranate resulted in the identification of 897 and 168 SSR markers corresponding to pri-miRNAs and pre-miRNAs, respectively (Patil et al., 2020). In *Medicago truncatula* genome, Min et al. (2017) identified 189 miRNA-SSRs in pri-miRNA sequences extracted from 356 non-redundant (NR) miRNAs. A similar analysis in the *Arabidopsis* genome identified 147 miRNA-SSRs from 169 pre-miRNA transcripts (Kumar et al., 2017). Since, presence of SSRs in these miRNA coding regions creates enormous possibilities for the development of predictive DNA markers for important phenotypes regulated by miRNAs (Patil et al., 2020). Also, the availability of information on potentially novel miRNA candidates for seed type trait as reported by Luo et al. (2018). Here, we performed preliminary study to develop seed type specific miRNA-SSRs and target gene based EST-SSR markers for future genomic applications. Which can aid in discover of master miRNA through association analysis or candidate gene based genome editing application for genetic improvement of seed type trait in pomegranate.

## Materials and Methods

### *In silico* Analysis and Identification of miRNA Coding Sequences

A total of 761 potential novel pre-miRNAs reported earlier by Luo et al. (2018) for seed type trait in pomegranate were retrieved. Firstly, all these sequences were searched for the presence of SSRs using MISA web tool (Beier et al., 2017; http://misaweb.ipk-gatersleben.de/misa/). We removed protein-coding sequences from pre-miRNA sequences through BLASTx against NR protein database (Altschul et al., 1997). Homology search was performed by using nc pre-miRNAs as query for BLASTn against Dabenzi genome (mismatch <1, with no gap and e-value ≤ 0.01) (Qin et al., 2017). The resulting contigs with flanking sequences of ∼800 bp around each query sequences were retrieved. Contigs were then examined for pri-miRNA regions using sequence-structure motif base: pre-miRNA prediction webserver **(** http://www.regulatoryrna.org/webserver/SSMB/pre-miRNA/home.html **)**. The contigs with no miRNA regions were excluded from the analysis. Finally, contigs with true pri-miRNAs and their pre-miRNA sequences were used for SSR survey.

### Designing of SSRs Specific to Pri- and Pre-miRNAs

SSR motifs were searched by keeping a minimum repeat length of 12 bp and defining 12, 6, 4, 3, 3 and 2 for mono, di, tri, tetra, penta and hexa nucleotide, respectively. The two SSRs interrupted within 100 bases were defined as compound SSRs. MISA statistics as obtained was used to draw frequency distribution graphs using Microsoft Excel. Batch Primer three v1.0 (https://wheat.pw.usda.gov/demos/BatchPrimer3) was used to design SSRs present in pri- and pre-miRNA sequences. All SSR primer pairs were designed to generate 100–400 bp amplicons with other specific parameters like: primer length (bp) 18–20 bp with 19 bp as optimum; GC content (%) 40–60, with the optimum value being 50% and Tm (°C) 52–60, with 55 as the optimum were used. For designing of EST-SSR primers Krait software was used with default parameters.

### ePCR Validation and Localization of miRNA-SSRs on Chromosomes

To check the amplification efficiency and locate the newly designed miRNA-SSRs on Tunisia genome, ePCR was performed using GMATA (Genome-wide Microsatellite Analyzing Tool Package) software (Wang and Wang, 2016) using algorithm (Schuler, 1997). A physical map with the SSR loci was constructed using MapChart v 2.2 software (Voorrips, 2002). MiRNA-SSR primers producing one to two alleles in Tunisia genome were then evaluated in three draft genome assemblies (Dabenzi, Taishanhong and AG 2017) through ePCR. The amplicon sizes were recorded for 80 miRNA-SSRs across the four pomegranate genomes using GMATA. The marker parameters were computed using GenAlEx v.6.5 (Peakall and Smouse, 2012).

### PCR Amplification and Diversity Analysis

Genomic DNA was extracted from the fresh leaf samples of 16 pomegranate genotypes (Supplementary Table S1) following the modified CTAB method (Ravishankar et al., 2000). For PCR, 32 pre-miRNA-SSR primers was initially synthesised and screened on a subset of six pomegranate genotypes *i.e.* Ganesh, Mridula, Jyoti, Yercaud, Kalpitiya and Co-white, following touch down PCR program with Prime-96™ Thermal Cycler (Himedia, India). Specific amplicons were confirmed by separating fragments on 3% metaphor gels. Subsequently, resulting subset of 10 informative miRNA-SSRs were selected for genetic diversity study in 16 pomegranate genotypes. For PCR experiments amplification was carried out in 10 μL reaction volume containing 1.0 μL of 10X PCR buffer, 1 μL (1 mM dNTP mix), 0.5 μL each of forward and reverse primers (10 pmol), 0.2 μL of *Taq* DNA polymerase 5U/μl (Himedia, India) and 1 μL (10 ng) of template DNA. Touchdown PCR was performed with the following conditions: 94°C for 5 min, followed by 16 cycles of 94°C for 30 s, decrease 0.2°C/cycle from 60°C for 30 s, 72°C for 45 s; followed by 25 cycles of 94°C for 30 s, 55°C for 30 s, 72°C for 45 s and a final extension at 72°C for 5 min. The amplified fragments were resolved on 3% metaphor agarose gels accompanied by visualization and documentation using a gel documentation system (Vilbert Dourmet, France). We computed genetic diversity parameters from the SSR genotyping data using GenAlEx v. 6.5 (Peakall and Smouse, 2012). The genetic cluster analysis was performed with the UPGMA (Unweighted pair group method with an arithmetic mean) method of NTSYS-pc v. 2.11 (Rohlf, 2000).

### Prediction of Potential Gene Targets for miRNAs

To elucidate the biological roles of selected pre-miRNAs, first homology search was performed against miRBase to determine their miRNA families. Then, target analysis for selected mature miRNAs was performed against 29,854 annotated gene models (mRNAs) reported for Tunisia genome (Luo et al., 2020). Prior to target analysis, CD-HIT tool with default parameters was used to reduce the redundancy (Li and Godzik, 2006), resulting in 21,877 unique sequences. Target analysis of mature miRNAs of 44 pre-miRNAs against 21,877 Tunisia gene models was performed using TAPIR (http://bioinformatics.psb.ugent.be/webtools/tapir; Bonnet et al., 2010). Blast2GO 6.0 (https://www.blast2go.com/blast2go-pro) was used to perform functional annotations of target genes for Gene Ontology (GO) and KEGG enrichment analysis, and Web Gene Ontology Annotation Plot was drawn using WEGO 2.07 (Ye et al., 2018). Further, based on the TAPIR alignment duplex target score (with cut off value ≤ 0.4) and duplex free energy ratio of hybridization (with cut off value ≥ 0.7) between miRNA/mRNA the regulatory networks were built using Gephi 0.9.2 software (Bastian et al., 2009). TAPIR target score considers the number of mismatches, gaps (introduced by bulges and loop structures) and number of G-U pairs located in position 2nd and 12th of 5’ seed regions of miRNAs to the target mRNA. Whereas the duplex miRNA-mRNA free energy ratio (Allen et al., 2005), is the ratio of the free energy of duplex to the free energy of the same duplex having only perfect matches.

## Results

### Characterization of miRNA-SSRs for Seed Type Trait

**FIGURE 1:**
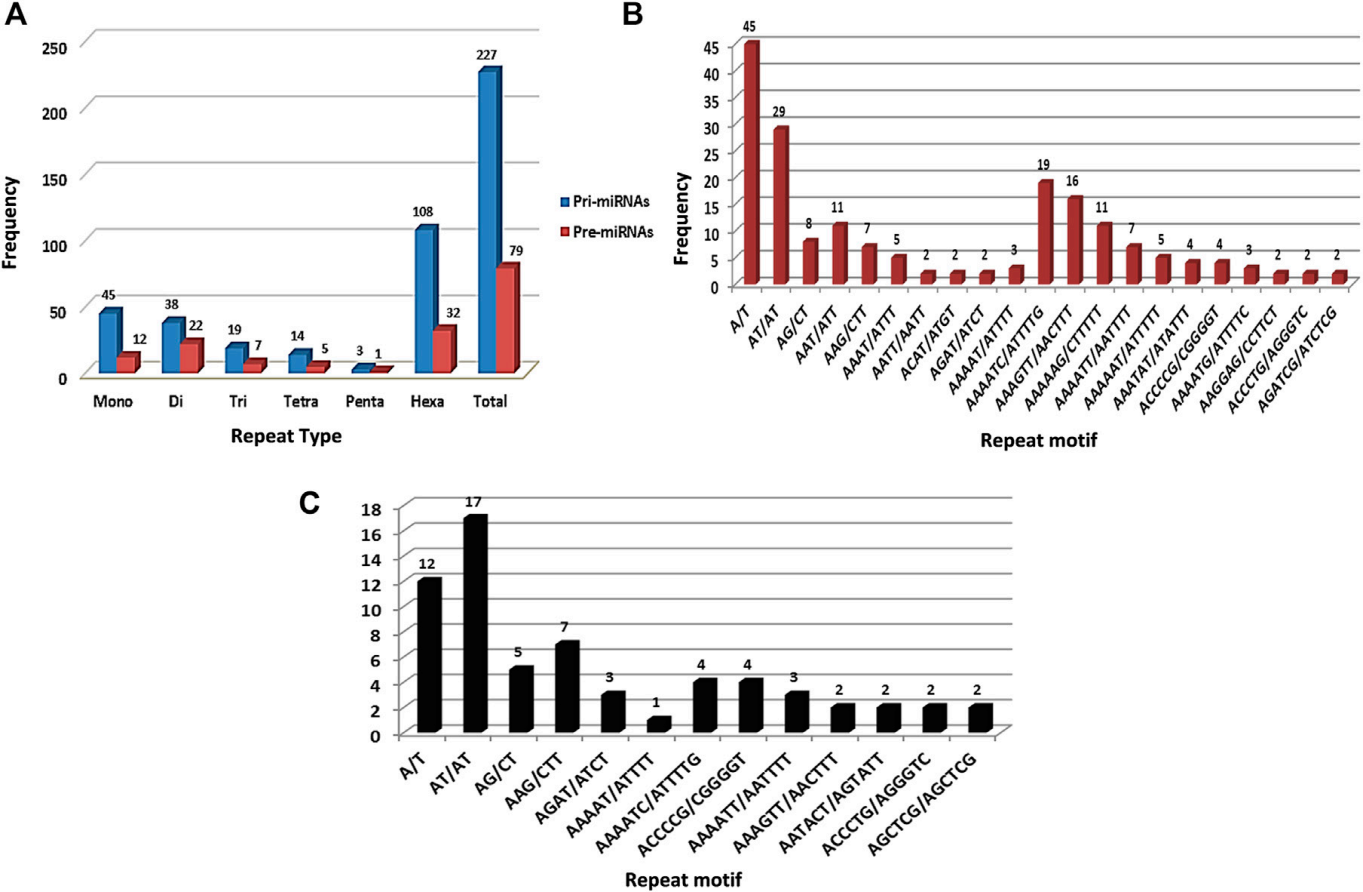
Frequency distribution for SSR repeats in pri- and pre-miRNA sequences of pomegranate genome cv. Dabenzi.

SSR survey of 761 pre-miRNA sequences facilitated detection of 199 SSR motifs corresponding to 144 pre-miRNAs. These sequences were then used for BLASTX search to remove protein-coding sequences. Resulting set of 69 non-coding pre-miRNA served as query sequences in BLASTn search against pomegranate genome cv. Dabenzi. A total of 69 highly-homologous contigs with ∼800bp flanking sequences around pre-miRNA complementarity regions were extracted. MiRNA prediction tool identified 63 contigs (∼821) that harboured pri-miRNAs and encoding 213 pre-miRNAs. SSR survey of these sequences using MISA identified 227 SSR motifs specific to 60 (95.2%) pri-miRNA and 79 motifs to 65 (30.5%) pre-miRNA sequences (Table 1). The pri-miRNA and pre-miRNA sequences represented 81.7 Kb and 22.2 Kb of pomegranate genome, respectively. The distribution frequencies of one SSR locus per every 0.36 and 0.28 kb were observed for pri- and pre-miRNAs, respectively. Total 55 pri- (87.3%) and 12 pre-miRNAs (5.63%) showed more than one SSR motifs. Out of 227 SSR motifs specific to pri-miRNAs and 79 to pre-miRNAs, 67 (29.5%) and 14 (17.7%) respectively were of compound type. Concerning the abundance of SSR motifs, hexa-nucleotides were the most pronounced (47.58%) followed by mono- (19.82%) and di-nucleotides (16.74%) in pri-miRNAs (Figure 1A). Similar SSR distribution pattern was observed in pre-miRNAs. In addition to this, pri-miRNAs revealed more abundance of A/T (100%) repeats followed by AT/AT (76.31%), which is also witnessed in pre-miRNA sequences (Figure 1B,C).

**TABLE 1:** Characterization of SSRs in pri- and pre-miRNA sequences of pomegranate genome.

Parameters	Pri-miRNA	Pre-miRNA
Number of sequences examined	63	213
Examined sequences size (bp)	81,788	22,214
Total number of identified SSRs	227	79
Number of sequences with SSRs	60	65
Number of sequences with more than 1 SSRs	55	12
Number of compound SSRs	67	14

Note* Pre-miRNAs: Precursor miRNAs; Pri-miRNAs: Primary miRNAs.

### Designing of miRNA-SSRs, ePCR Validation and Mapping on Chromosomes

We designed 132 primers for SSRs located within 60 pri-miRNA sequences. The details of primer pairs are given in (Supplementary Table S2). Out of 132 miRNA-SSR primers, 77 (58.33%) primers were designed targeting hexanucleotide repeats; followed by 30 (22.73%), 12 (9.09%), 10 (7.57%) and 3 (2.27%) for di, tri, tetra and penta nucleotide repeats, respectively. However, 46 (34.85%) primers were specific to pre-miRNA sequences.

To assess amplification efficiency, specificity and chromosome locations of the miRNA-SSRs, we performed ePCR or e-mapping of SSRs on the chromosomes of Tunisia genome. As a result, 123 of 132 SSRs were successfully got validated across eight chromosomes, producing alleles of single, two, three or more than three alleles in the Tunisia genome (Table 2). However, nine primers did not map to Tunisia chromosomes. Total 26 (21.14%) primers produced single amplicons, whereas 54 (43.90%) primers yielded two alleles and 43 primers had ≥3 alleles when assayed across the chromosomes.

**TABLE 2:** Experimental validation of 123 miRNA_SSRs in Tunisia genome in comparison to other three draft genomes of pomegranate cultivars Dabenzi, Taishanhong and AG2017 through ePCR or eMapping.

	ePCR validation of 123 miRNA_ SSRprimers for genotyping applications																			
	Tunisia genome					Dabenzi genome					Taishanhong genome					AG2017 genome				
	Allele No					Allele No					Allele No					Allele No				
	one	two	three	>three	TNP	one	two	three	>three	TNP	One	two	three	>three	TNP	one	two	three	>three	TNP
**Chm_1**	1	6	5	—	12	1	7	4	—	12	1	6	5	—	12	1	4	6	—	11
**Chm_2**	7	16	13	—	36	5	15	16	—	36	3	19	13	—	35	4	12	14	2	32
**Chm_3**	2	4	5	—	11	1	5	4	1	11	0	6	5	—	11	1	4	2	3	10
**Chm_4**	2	8	3	—	13	1	7	5	—	13	4	6	3	—	13	1	6	4	1	12
**Chm_5**	2	5	3	—	10	1	5	4	—	10	1	6	3	—	10	0	4	5	—	9
**Chm_6**	5	5	3	2	15	3	7	5	—	15	3	4	5	3	15	4	7	1	—	12
**Chm_7**	5	6	4	—	15	0	9	6	—	15	4	7	4	—	15	3	6	4	—	13
**Chm_8**	2	4	5	—	11	2	3	5	1	11	1	5	4	—	10	1	5	3	2	11
Total	26	54	41	2	123	14	58	49	2	123	17	59	42	3	121	15	48	39	8	110

Note* Chm: chromosomes; TNP: total number of primers.

**FIGURE 2:**
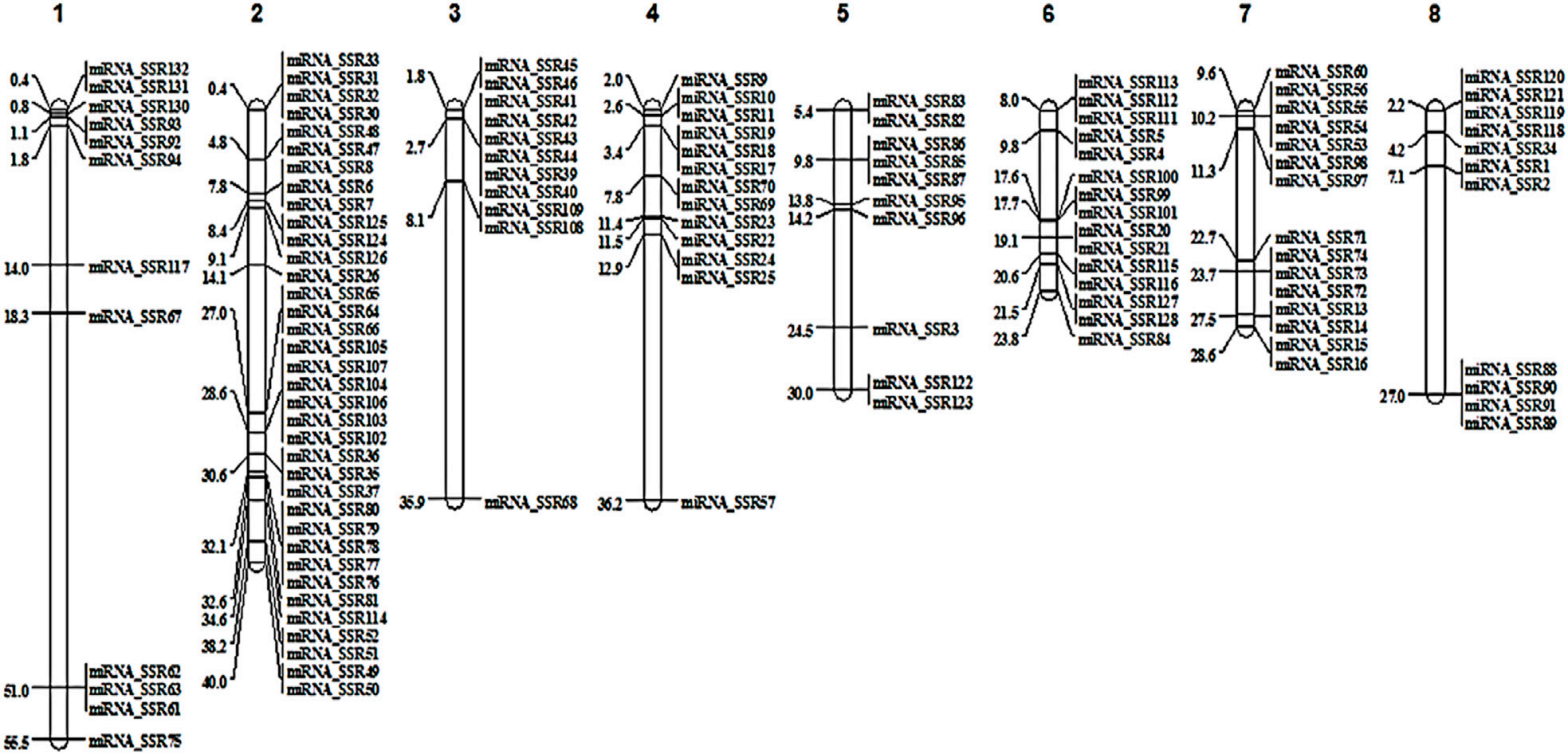
Chromosome specific localisation of miRNA-SSR markers on Tunisia genome.

Physical locations of miRNA-SSRs were visualized on eight chromosomes. A total of 123 markers were mapped onto individual chromosomes of Tunisia, of which Chm_2 (36 markers), Chm_6 (15) and Chm_7 (15) had the higher number of SSRs, followed by Chm_4 (13), Chm_1 (12), Chm_3 (11), Chm_8 (11) and Chm_5 (10) respectively. Figure 2 illustrates the chromosome-wise locations (Mb) of all the miRNA-SSRs. The SSR map presented here would enable accurate choice of informative miRNA-SSRs based on their genomic positions (Supplementary Table S3).

### Identification of Informative miRNA-SSRs Through ePCR Across the Pomegranate Genomes

We confirmed amplification of 123 miRNA-SSRs across three assemblies of pomegranate genome *i.e.* Dabenzi, Taishanhong and AG 2017. As a result, 80 SSRs were identified that produced one to two alleles across three genomes including Tunisia (Table 3). Further, the amplicons of these genotypes were recorded to compute various marker parameters (Supplementary Table S4). Of the total 80 SSRs, 77 (96.25%) showed polymorphism across four genotypes. The assay generated a total of 213 alleles spanning eight chromosomes. The Na per locus ranged from 2 to 6, with an average value of 2.66 alleles/loci. The MAF per locus varied between 0.25 and 0.88, with an average of 0.58. The PIC values ranged from 0.25 to 0.93, with an average of 0.58. In the present dataset, 63 SSRs had the PIC values ≥ 0.50, implying their highly informative nature. The average Shannon information index was 0.82 for the four genomes tested.

**TABLE 3:** Chromosome specific marker statistics for 80 miRNA-SSR primers assayed through ePCR across the four pomegranate genotypes based on their genome sequences.

Chromosome	TNP	TPP	Na	MAF	Ne	*I*	*Ho*	*He*	PIC
**Chm_1**	7	7	19 (2.71)	0.55	2.27	0.87	0.89	0.54	0.63
**Chm_2**	23	23	65 (2.83)	0.55	2.30	0.89	0.78	0.54	0.62
**Chm_3**	6	6	15 (2.50)	0.59	2.07	0.79	0.76	0.51	0.58
**Chm_4**	10	9	24 (2.40)	0.60	2.08	0.74	0.80	0.47	0.54
**Chm_5**	7	7	18 (2.57)	0.57	2.20	0.84	0.79	0.53	0.61
**Chm_6**	10	8	22 (2.20)	0.66	1.82	0.62	0.63	0.40	0.45
**Chm_7**	11	11	30 (2.73)	0.61	2.03	0.80	0.73	0.49	0.56
**Chm_8**	6	6	20 (3.33)	0.52	2.83	1.03	0.83	0.58	0.67
Total/mean	80	77	213 (2.66)	0.58	2.20	0.82	0.78	0.51	0.58

Note* Chm: Chromosome; TNP: total number of primers; TPP: total number of polymorphic primers; Na: Numbers of alleles; MAF: major allelic frequency; Ne: Number of Effective Alleles; *I*: Shannon’s Information Index; *Ho*: Observed heterozygosity; *He*: Expected heterozygosity; PIC: polymorphic information content.

### Functional Classification and Pathway Enrichment Analysis for miRNA Target Genes

To assign functional roles to the identified miRNA-SSRs, we carried out target analysis using 21,877 unique gene models from Tunisia genome. This resulted in identification of a total of 2,306 targets, of which 1935 were found unique targets and 371 as common targets (Supplementary Table S5). The predicted targets belonged to 24 miRNA families, with -MIR156b having the highest targets (655), followed by ath-MIR5655 (592), MIR5021_1 and 2 (522), ath-MIR5651_3 (394), ath-MIR157c (43) and ath-MIR5651_1 (39). Further, based on lowest target score (≤4) and highest minimum free energy (Mfe) ratio of hybridization (≥0.7), 2,306 targets were narrowed down to a set of 754 candidate genes, which were negatively regulated by 24 miRNA families. Target analysis led to the identification of five informative miRNA-SSRs *i.e.* miRNA_SH_SSR69, miRNA_SH_SSR36, miRNA_SH_SSR103, miRNA_SH_SSR35 and miRNA_SH_SSR53 influencing expression of multiple genes serving as transcription factors, enzymes and transporters involved in seed development and maturation to impart seed type (Supplementary Table S6).

### Gene Ontology Analysis

**FIGURE 3:**
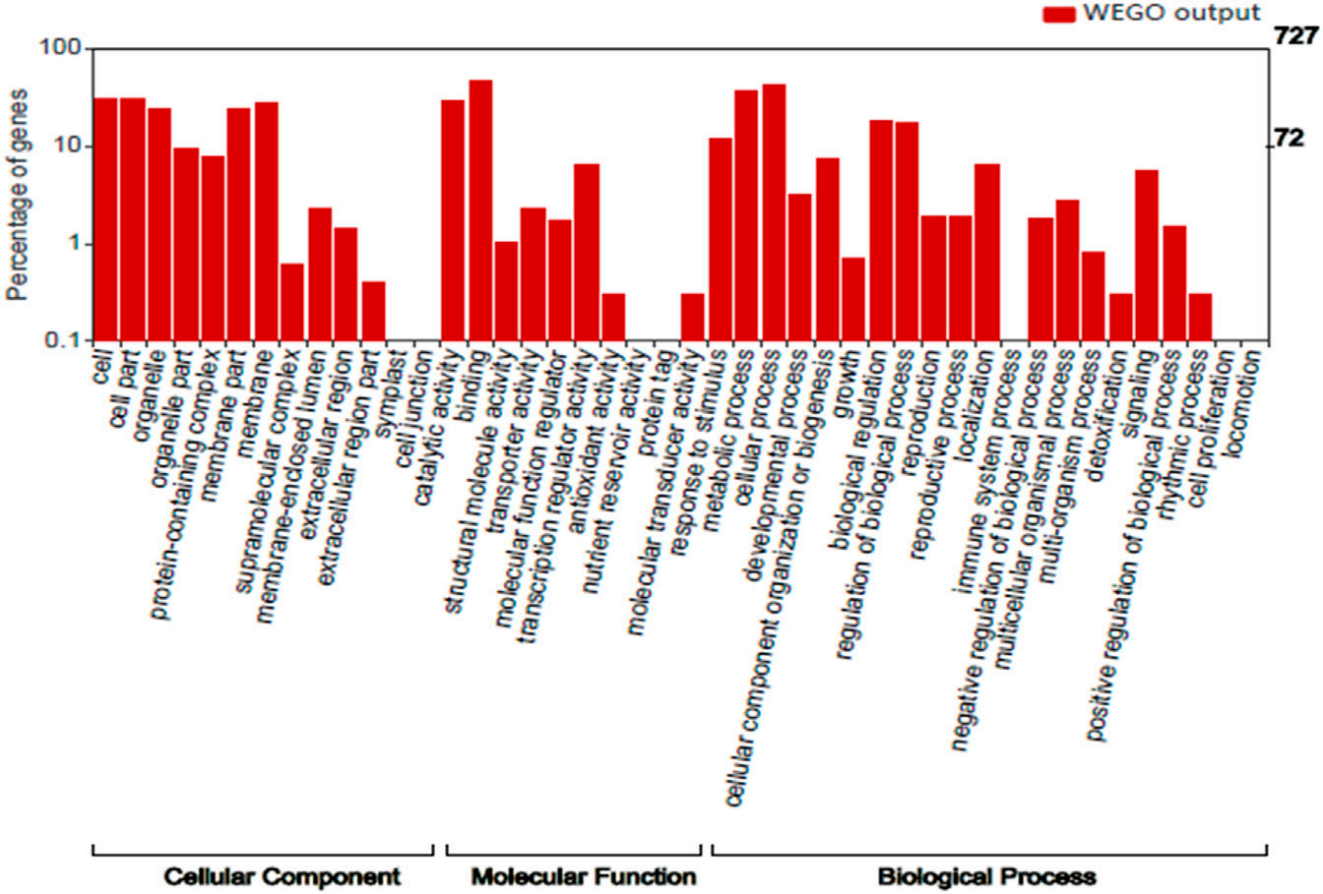
Distribution of GO terms in the Cellular component, Molecular function and Biological process category.

We performed Gene Ontology for 727 top genes targeted by miRNAs to find the potential contributions of these genes during seed development and maturation. The target genes were grouped into three classes: biological process (20 GO terms), molecular function (11 GO terms), and cellular component (13 GO terms) (Figure 3). GO analysis showed most of the genes were found to be associated with biological process like metabolic process, cellular process, biological process regulations, cellular component organization or biogenesis, localization and signaling. With respect to cellular components, many genes are part of cell, organelle and parts, protein containing complex and membrane. However, with respect to molecular functions many genes have role of binding and catalytic activity, transcription regulator activity, transporter activity and response to stimulus. Therefore, the GO analysis clearly showed the role of miRNA-targeted genes in seed development and maturation.

**FIGURE 4:**
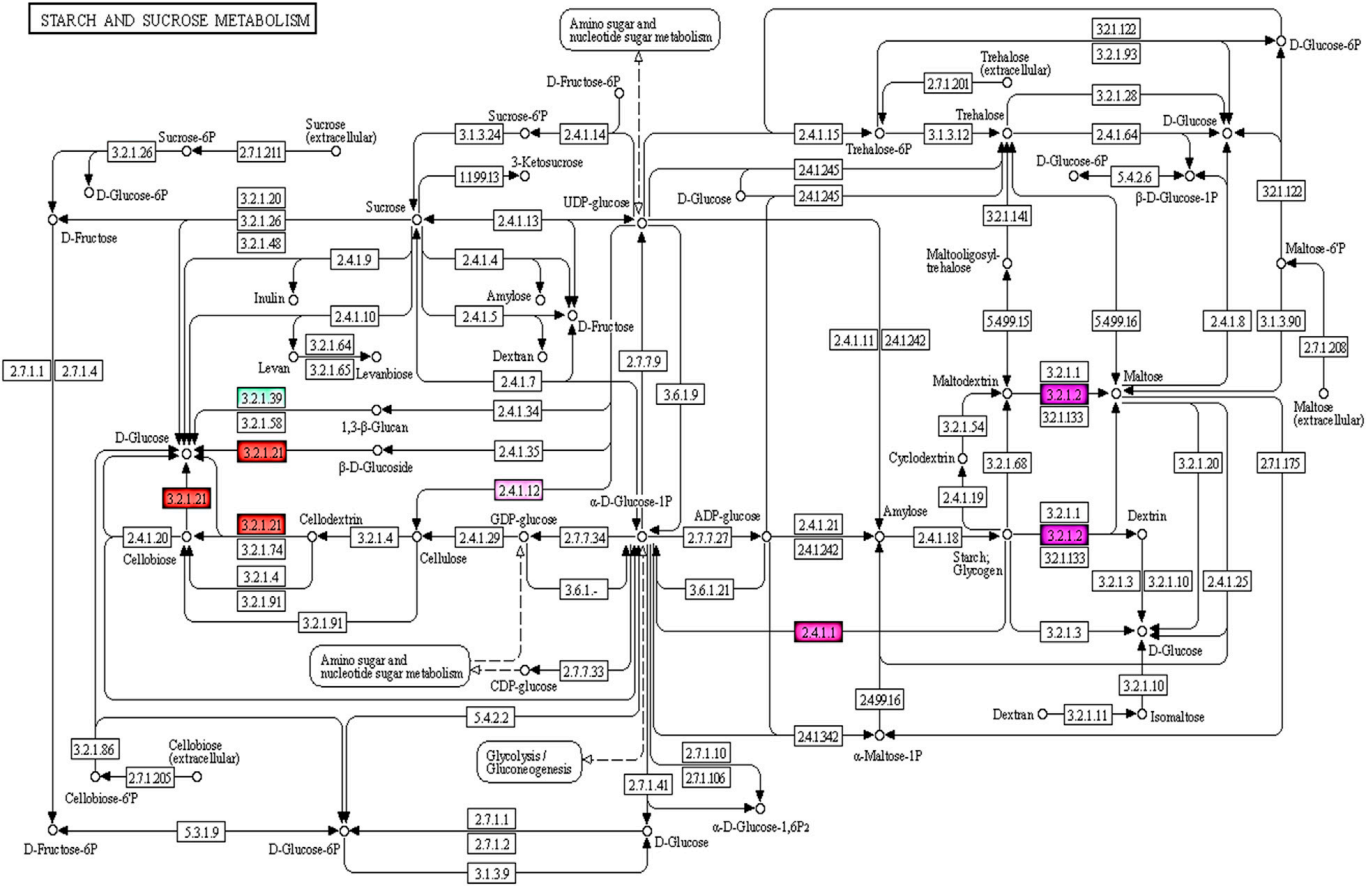
KEGG pathway showing enzymes which are targeted by miRNAs involved in starch and sucrose, amino sugar and nucleotide metabolism of seed development.

The KEGG pathway enrichment analysis revealed 95 of 727 targets code for 101 enzymes involved in 58 pathways, with the highest representation from Starch and sucrose metabolism, Glycerophospholipid metabolism, Glycerolipid metabolism, Amino sugar and nucleotide sugar metabolism, Pentose and glucuronate interconversions and so forth (Supplementary Table S7). Among these pathways maximum of seven enzymes were part of starch and sucrose metabolism & four enzymes involved in Amino sugar and nucleotide sugar metabolism which is depicted above *i.e.* ec:3.2.1.39 -endo-1,3-beta-D-glucosidase, ec:2.7.7.27-adenylyltransferase, ec:3.2.1.26- invertase, ec:2.4.1.12 - synthase (UDP-forming), ec:3.2.1.21-gentiobiase, ec:3.2.1.2-saccharogen amylase, ec:2.4.1.1-phosphorylase, ec:1.1.1.22-6-dehydrogenase, ec:3.2.1.55-end alpha-l-arabinofuranosidase,ec:2.4.1.43-4-alpha-galacturonosyltransferase (Figure 4).

### Construction of miRNA-Mediated Regulatory Networks

**FIGURE 5:**
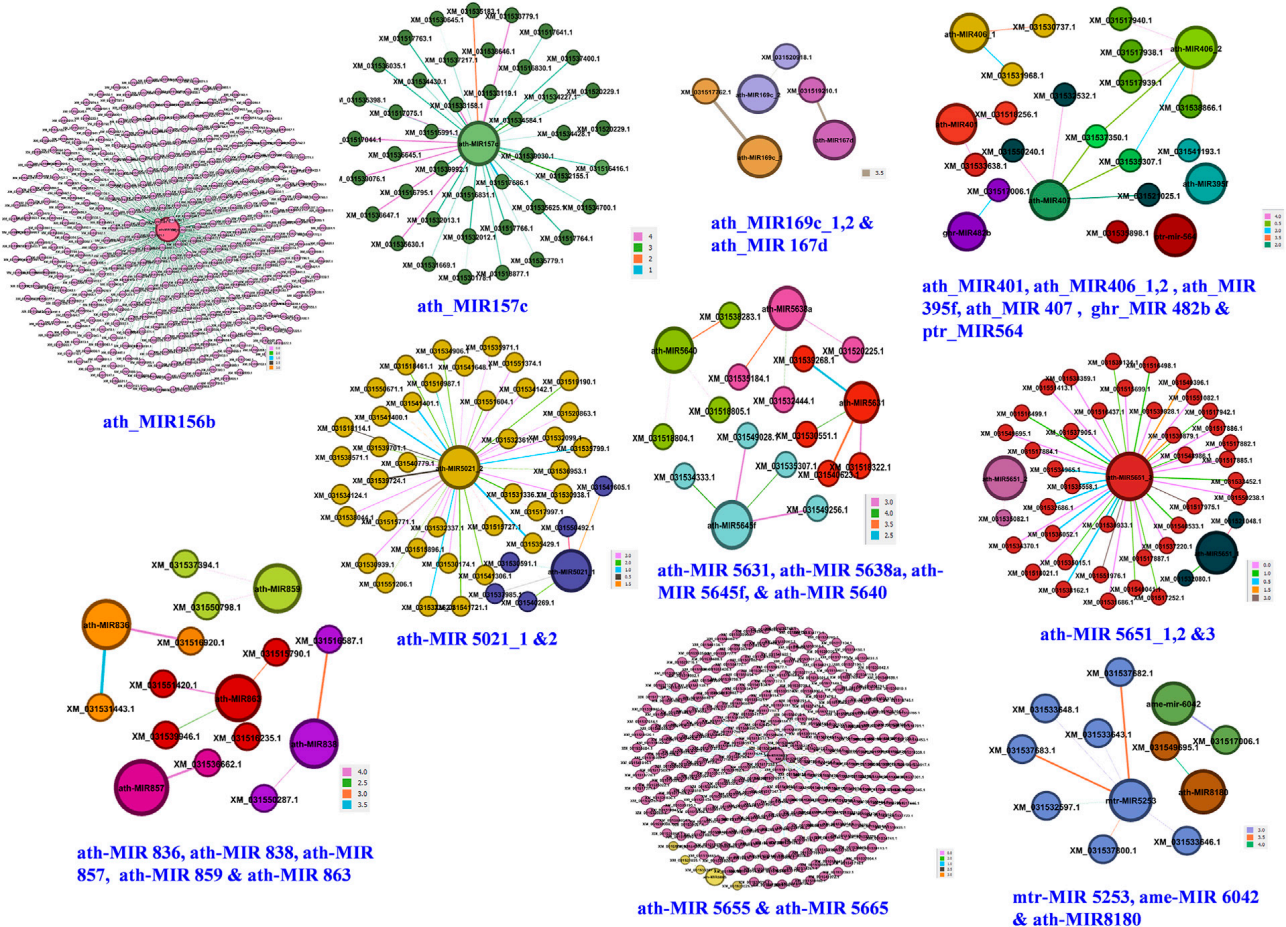
Genes regulatory network for 24 miRNA families. Darker lines with color codes for each family indicate stronger interactions of miRNAs with their target genes based on highest minimum free energy ratio of hybridization and lowest alignment scores.

Ten independent networks were obtained for 24 miRNA families targeting 754 candidate genes with lowest target score and highest Mfe (Figure 5). The ath-MIR156b had maximum targets (403 genes), followed by ath-MIR5655 (248 genes), ath-MIR5651_3 (38 genes), ath-MIR5021_2 (19 genes) and ath-MIR157c (9 genes). Several other miRNAs had one to three targets. Network graphs depicted the candidate genes involved in seed development and their regulation by the different miRNA-families (Supplementary Table S8).

### Developing EST-SSRs From Target Genes

To provide markers for selecting seed type trait, SSR survey of 1935 unique target genes facilitated in identification of 7,688 perfect SSR motifs, with highest frequency of hexa-nucleotide repeats (3,497), followed by di- (1,632) and tri-nucleotides (1,661). Penta-nucleotides (181) were the least frequent followed by mono-nucleotides (276) (Supplementary Figure S1). Finally, a total of 413 functional EST-SSR markers were designed, majority of which (204) targeted hexa-nucleotide motifs accompanied by di- (95), and tri-nucleotides (73). These EST-SSRs represent important genomic resources that would enable discovery of genes/QTLs for seed type traits in pomegranate (Supplementary Table S9).

### PCR Based Validation and Diversity Analysis

We synthesized a set of 32 pre-miRNA-SSRs specific to seed type and assayed these selected SSRs on six pomegranate genotypes. As a result, 28 (87.5%) miRNA-SSRs yielded the amplicons of expected size, whereas no amplification was recorded for four miRNA-SSRs. Of these, 25 (89.29%) miRNA-SSRs revealed polymorphism across six pomegranate genotypes, while remaining three markers (MIR_SH_SSR41, 69 and 90) were monomorphic (Supplementary Table S10). Marker profiles of pomegranate genotypes using selected SSRs (Supplementary Figure S2).

The 28 primers produced one to two alleles, with the PIC values ranging between 0 and 0.54. Based on these results a subset of 10 miRNA-SSRs was selected and genotyped on 16 pomegranate genotypes to estimate genetic diversity. These markers amplified total 22 alleles with average of two alleles per locus. The MAF per locus ranged from 0.50 to 0.87, with average of 0.70. The *He* ranged from 0.23 to 0.50, with an average of 0.39. The PIC values ranged from 0.24 to 0.52, with an average of 0.40 (Supplementary Table S11). The mean Shannon’s information index value of 0.57 was observed among the genotypes.

**FIGURE 6:**
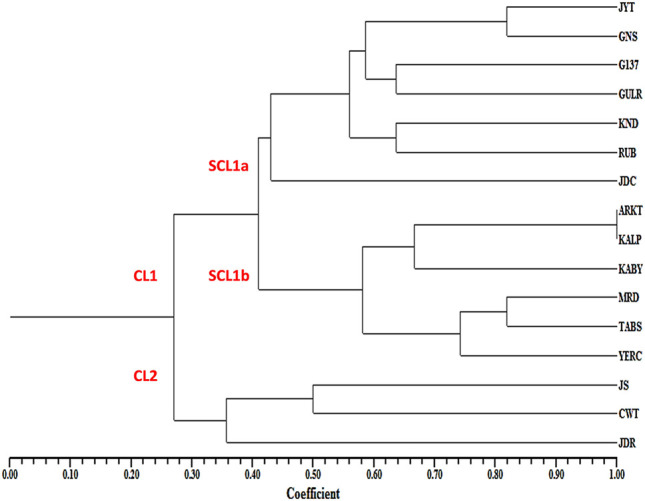
Dendrogram showing the genetic relationships among 16 pomegranate genotypes based on ten MIR_SH_SSR markers.

In the UPGMA tree, all the 16 pomegranate genotypes were grouped into two major clusters, with cluster one harboring 13 and cluster two containing three pomegranate genotypes. The cluster one was further divided into two sub clusters 1a and 1b (Figure 6).

## Discussion

Recent advances in DNA sequencing and computational analysis in pomegranate have facilitated large-scale mining of DNA markers including EST-SSRs (Ophir et al., 2014), EST-SNPs (Harel-Beja et al., 2015), genomic SSRs (Liu et al., 2020), and most recently miRNA-SSRs (Patil et al., 2020). This provided immense opportunities to integrate information from genome, transcriptome and non-coding RNAs to understand genetic mechanisms that are regulating important traits in pomegranate. The DNA markers present in miRNAs have potential utility for the identification of master miRNAs that regulate the key genes for fruit quality traits (Patil et al., 2020). Here, we provided novel pre-miRNAs reported for seed type trait in pomegranate (Luo et al., 2018), to develop, validate and localization of miRNA-SSRs markers on Tunisia chromosomes. Also report development of 413 functional EST-SSRs, which may be of potential use for functional validation for seed type and breeding applications.

### Survey of SSR Motifs in the miRNA-Coding Sequences

SSR survey in pri-miRNAs resulted in designing of 132 miRNA-SSR primers, of which 46 primers are specific to pre-miRNAs. Previously, we reported genome-wide survey for miRNA-SSRs for seedling to fruit development stages in pomegranate genome using *in silico* approaches. Homology-based search has enabled development of large-scale miRNA-SSRs in several plant species including *Medicago truncatula* and *Arabidopsis thaliana* (Kumar et al., 2017; Min et al., 2017). In the present study, frequency distribution analysis of SSR occurrence in both pri-and pre-miRNAs revealed the abundance of hexa-nucleotides repeats followed by mono-and di-nucleotides. With respect to motif types, we found A/T repeats were the most abundant, followed by AT/AT (76.31%) in pri- and pre-miRNA sequences. These results remain in good agreement with our previous finding as reported in pomegranate (Patil et al., 2020). By contrast, mono- and di-nucleotide repeats were pronounced in miRNAs coding sequences of *M. trucatula*, *A. thaliana* and other species (Chen et al., 2010; Kumar et al., 2017; Min et al., 2017).

### Functional Classification and Pathway Enrichment for miRNA Target Genes

Mining of miRNA-SSRs and use of its mature miRNA for target prediction followed by functional annotations could lead to identification of candidate genes underlying traits of interest. Therefore, miRNA-SSRs (132) and EST-SSRs (413) derived from miRNA-coding sequences and their 1935 unique gene targets, respectively have greater implications for improving seed type trait in pomegranate. Our findings on target gene functions are congruent with the previous reports in pomegranate based on an integrated analysis of microRNA identification and mRNA expression profiling for seed type trait (Luo et al., 2018). The study reported 408 and 335 mRNA targets in the Tunisia and Sanbai genomes at 60 and 120 DAF, respectively (Luo et al., 2018). Xue et al. (2017) also reported 34,221 genes that had differential expression (DEGs) between soft and hard-seeded pomegranate using global gene expression profiling. As elucidated from the GO annotations, the functional roles of the miRNA gene targets remain in strong agreement with our present study.

In the present study, KEGG pathway enrichment analysis uncovered possible pathways associated with hard and soft seeded types in pomegranate. Cao et al. (2015), through comparative proteomics analysis identified key enzymes involved in tricarboxylic acid cycle (TCA) and mitochondrial metabolism during fruit maturation periods in soft seeded pomegranate cultivar Zhongnonghong. Recently, Luo et al. (2018) reported 14 and 8 DEG targets for miRNAs between soft and hard-seeded pomegranate at 60 and 120 DAF, which are part of 18 and eight pathways. Similarly, Xue et al. (2017) also reported DEGs between soft and hard-seeded pomegranate that participate in photosynthesis, benzene propane synthesis, phospholipid metabolism, ribosome metabolism and ubiquitin mediated proteolysis. Another study in pomegranate revealed genes targets of 41 miRNA families that were associated with in 107 major pathways controlling fruit development.

Here, we found 101 enzymes that are part of 58 pathways, seven enzymes participating in starch and sucrose metabolism, whereas four enzymes had roles in amino sugar and nucleotide sugar metabolism. Niu et al. (2018) found that ovules-to-seed transition in pomegranate was regulated by the co-expression of many proteins in the short term. The authors predicted protein-protein interactions among 505 and 549 proteins at 60 and 120 DAF, respectively. Of these, seven proteins were involved in phenylpropanoid biosynthesis whereas 15 had roles in starch and sucrose metabolism. Research suggests a close association between UDP-glucose pyrophosphorylase (UGP) and sucrose synthase (SUS) with cellulose biosynthesis in plants (Andersson-Guunneras et al., 2006). Similarly, differences in expression abundance at both gene and protein levels between Tunisia and Sanbai suggested that UGP2 and SUS3 were upregulated at 60 DAF and downregulated at 120 DAF in Tunisia (Niu et al., 2018). Evidence of lower lignin and higher cellulose during early fruit developmental stages supported the contradictory roles of lignin and cellulose in cell wall formation in soft-seeded pomegranate. Our findings are congruent with an earlier report of higher cellulose-related gene expression and cellulose content in soft-seeded pomegranate varieties in contrast with the hard-seeded varieties with higher lignin (Zarei et al., 2016). Its also may be due to fact that use of Tunisia gene models for target analysis in our study identified several targets engaged in cellulose synthesis.

### miRNA Regulatory Networks

MiRNAs are usually known to negatively regulate their targets. The complementarities between the miRNA and their target genes serves as a basis for identification of the gene targets engaged in a variety of processes to plant growth and development (Singh et al., 2017; Mishra and Bohra, 2018). Therefore, here we established regulatory networks among miRNA and mRNA to understand their roles in seed type. Previously, it was reported that seed type is related to cell wall biosynthesis (Zarei et al., 2016). Numerous candidates like MYB, WRKY, AP2-like, MYC and NAC are known to play important roles in pomegranate and hawthorns in regulating seed type. These transcription factors are involved in brassinosteroid biosynthesis, cell division, lignin, cellulose flavonoid and xyloglucan biosynthesis (Dai et al., 2013; Xue et al., 2017; Luo et al., 2020). In our study target and network analysis revealed ath-miR156b had maximum targets followed by ath-MIR5655, ath-MIR5651_3, ath-MIR5021_2 and ath-MIR157c suggesting these are the most abundant families in pomegranate. We propose a possible role of all these miRNA families in complex regulation of seed maturation since these showed strongest interactions with MYB, auxin response factors, WRKY and NAC, AP2/ERF and B3 domain-containing transcription factors and enzymes mainly involved in cellulose, lignin and sugar metabolism etc. However, Luo et al. (2018) reported mdm-miR164e- and mdm-miR172b-targets included WRKY, MYC and NAC1 mainly involving brassinosteroid biosynthesis, cell division and lignin biosynthesis. Xia et al. (2019) characterized the role of a NAC transcription factor (PgSND1-like) involved in the regulation of seed type in pomegranate. These findings suggest that a complex biological process mediated by miRNA–mRNA network controls pomegranate seed type (Luo et al., 2018).

Huang et al. (2013) found mir156 is the largest family while studying the miRNAs and their purgative targets in *Brassica napus* seed maturation. They found majority of the family members were primarily expressed in the embryo and they may also regulate the developmental transition to germination. In pomegranate, Luo et al. (2018) identified 40 miRNA families for seed type trait, with miR156 family showing the highest 31 members (14.98% of the total). Similar reports exist in pear (Wu et al., 2014) and apple (Xing et al., 2016). Saminathan et al. (2016) found miR157 as the most abundant family in pomegranate. Interestingly, we noticed fewer targets for ath-MIR157c in this study. The miR156 and miR157 are known to regulate ovule development by targeting SQUAMOSA-promoter binding protein or box transcription factors (SPL/SBP) in plants (Liu et al., 2017). Evidence supports the role of miR156/157 and miR172 in controlling flowering and the vegetative-to-reproductive transition in plants (Wu et al., 2009). Therefore, participation of miRNA156 family has been well documented during seed cycle *i.e.* during dormancy modulation, germination, development and maturation. Earlier research has shown a role of AP2-like TFs in regulating seed size and seed mass in *Arabdopisis* (Ohto et al., 2009).

In our previous report, we found ath-MIR5651 is strongly interacting with MYB-like transcription factors during fruit development in pomegranate (Patil et al., 2020). In brassica, Singh et al. (2017) predicted involvement of miR5021 in starch and sugar metabolism and the corresponding target glycosyl hydrolase family proteins that participate in pentose and gluconate inter-conversion pathway during abiotic stress. Borges et al. (2011) reported 25 potentially novel miRNAs including miR5021 processed in sperm cells and pollen. The authors found sub functionalization of these miRNAs in association with a putative germline-specific Argonaute complex. ARGONAUTE 5 (AGO5) localizing preferentially to the sperm cell cytoplasm in mature pollen, suggest possible role of these miRNAs in germline differentiates and formation of mature male gametophyte. Amiteye et al. (2013) discovered 109 *Boechera* small RNAs while unrevealing the role of miRNAs during switch from sexual to apomictic reproduction in *Boechera* species. They found these miRNAs showing significant homology to 407 *Arabidopsis thaliana* small RNAs including the *A. thaliana* pollen-specific ath-miR5021 indicating possible role in reproduction.

On the other hand, we have identified families that have only few targets *viz.,* ath-MIR167d, ath-MIR169c and ath-MIR401 had two targets each, *i.e.* zinc metalloprotease EGY2 and chloroplastic LOC116189522, kinesin-like protein NACK2 and protein transport protein SEC16A homolog, uncharacterized LOC116188785 and pentatricopeptide repeat-containing protein At5g08490, respectively. For ath-MIR395f we found one target *i.e.* pentatricopeptide repeat-containing protein At1g56570. The essential role for miRNA167 in maternal control of embryonic and seed development was confirmed by the deletion of four *microRNA167* (*MIR167*) genes in *Arabidopsis* using gene editing. Plants with *mir167a* mutant and the *ARF* overexpression were found defective in anther dehiscence and ovule development (Yao et al., 2019). Zhou et al. (2007) reported UV-B stress induced upregulation of miR401 in *Arabidopsis*. Kim et al. (2010) reported miR395c/e differentially affects seed germination of *Arabidopsis* under stress conditions.

Besides, the miRNA families that specifically targeted embryogenesis-associated protein, small heat shock protein and resistance proteins RPM1 and RPP13 were mtr-MIR5253, ptr-mir-564, ath-MIR8180 and ath-MIR5021_2. This implied towards that these miRNA families orchestrate the expression of important TFs and enzymes during the early stages of seed development and regulate proteins involved in storage compound synthesis and transport in the mature seeds. Heat shock proteins expressed during pomegranate fruit maturation periods implicate them in protecting the pomegranate seeds from adverse stress such as extreme temperatures (Cao et al., 2015). Similarly, development of miRNA regulatory networks has greatly helped expanding knowledge about the miRNA and their target gene interactions in various other crops *viz.,* bread wheat (Nigam et al., 2015), radish (Zhang et al., 2016), maize (Wu et al., 2016) and brassica (Singh et al., 2017). Finally, through target analysis we could identify few informative miRNA-SSRs that can be deployed after functional validation for genetic improvement of seed type.

### Experimental Validation and Mapping of miRNA-SSRs Having Associations With Seed Type

Despite availability of a diverse array of DNA marker systems in pomegranate, their applications in improvement programs remain limited, possibly due to lack of trait-associated markers (Patil et al., 2020). Mondal and Ganie (2014) reported miR172b-SSR that could differentiate rice genotypes with respect to their salt stress response. Therefore, such functional DNA markers hold great potential in pomegranate for trait mapping and genome editing applications.

E-mapping of 132 miRNA-SSRs on Tunisia chromosomes led the validation of 123 markers across all the chromosomes. A physical genetic map was developed based on the information on start positions of all the markers. Information on start positions of markers had allowed successful anchoring of the new SSR markers to the physical map of groundnut (Lu et al., 2019). Portis et al. (2016) also reported genomic distribution of SSRs and their relations with annotated genomic components (gene space) based on the information on assembled pseudomolecules of globe artichoke genome. The information on chromosome wise location of miRNA-SSR markers could help in precise validation and introgression of genes/QTLs for seed type in pomegranate. Several reports exist on fine mapping of genes/QTLs enabled by an SSR-based physical map (Zhao et al., 2017).

### Identification of Informative miRNA-SSRs Through ePCR

Through ePCR we identified 80 primers producing one to two alleles across the four pomegranate genomes. Out of these, 63 primers had PIC values greater than 0.50, implying their highly informative nature. Therefore, we believe that 63 highly polymorphic miRNA-SSRs identified in this study would be a valuable genomic tool for understanding the genetic makeup of seed type trait of pomegranate. Recently, Uncu and Uncu (2020)) developed chromosome anchored SSR markers for carrot genome assembly, and identified 51,160 single-locus markers through e-PCR. Further, they experimentally evaluated 50 markers across 17 carrot accessions and found 46 markers produced expected product sizes suggesting accuracy rate of 90% in predicting the amplification profiles by e-mapping. The DNA markers designed through *in silico* mining have been succefully validated through experimental assays in wheat (Han et al., 2015), cucumber (Liu et al., 2015), bitter gourd (Cui et al., 2017) and tobacco (Wang et al., 2018). Therefore, the dataset generated here assist pomegranate research community in future genetic research and applied breeding.

Apart from miRNA-SSRs, we report large scale development of 1935 unique target gene based 413 EST-SSR markers for mapping genes/QTLs for seed type trait in pomegranate. In a previous study, we reported a subset of 58 functional EST-SSR markers from 128 target genes (Patil et al., 2020). Similarly, miRNA-mRNA complementarity has permitted the development of SSR from predicted target genes in several other crops such as 700 SSRs from 621 target genes in *Brassica* (Singh et al., 2017).

### Wet Lab Validation and Diversity Analysis

Through wet lab experiments, we observed that 28 primers showed clear and reproducible amplifications on metaphor gels, with an average PIC value of 0.38. Earlier we found an average PIC value of 0.29 for 47 pre-miRNA-SSRs validated on eight pomegranate genotypes (Patil et al., 2020). A similar PIC value of 0.43 was obtained when 34 miRNA-SSRs were screened on six *Brassica* species (Fu et al., 2013). In our study, 13 miRNA-SSRs had higher PIC (≥0.48) on metaphor gels, implying towards their greater potential for future research on seed type trait in pomegranate. According to Bandelj et al. (2004), DNA markers having PIC values greater than 0.5 can be considered informative.

Twenty two alleles were obtained by analysing 10 miRNA-SSRs on 16 pomegranate genotypes. However, 87 alleles obtained for 15 miRNA-SSRs in our earlier study on 18 pomegranate genotypes using fragment analyser indicated that SSR fragments can be better separated using high resolution systems (Patil et al., 2020). The gel detection system used in the present study has allowed detection of a relatively less number of alleles and lower PIC values, possibly due to its poor resolving power. Hence we advocate that an allele detection system with a higher resolving power such as based on capillary electrophoresis would be more appropriate for future research using these new SSR markers. In our study, the higher diversity index (0.62–0.69) and PIC values (0.44–0.52) were observed for five MIR_SH_SSRs 11, 23, 26, 37 and 64 suggesting highly informative in nature. The mean Shannon’s information index indicated moderate genetic diversity level among the 16 genotypes assayed. These results are in agreement with earlier finding as reported for miRNA-SSRs in pomegranate (Patil et al., 2020).

Cluster analysis separated 16 pomegranate genotypes into two major clusters. The cluster one constituted two sub clusters, where most of the soft seeded genotypes were clearly separated from hard seeded types with few exceptions. Sub-cluster 1a is composed of seven soft seeded cultivars (Jyoti, Ganesh, G137, Gulesha Red, Kandhari, Ruby and Jodhpur collection). Similarly, sub cluster 1b contained four hard-seeded types (Kalpitiya, Kabuli Yellow, Tabesta and Yercuad) with a slight deviation. Cluster two had three pomegranate cultivars belonging to soft seeded (Jallore Seedless) and hard seeded cultivars (Co-white and Jodhpur Red). These results indicated that the seed type specific miRNA-SSRs developed here could differentiate the genotypes according to their seed trait. Sequence variations in miRNA precursor molecules can have profound impact on the expressions of the genes associated with varietal seed type trait in pomegranate. For instance, Joy et al. (2018) highlighted the significance of SSRs in pre-miRNAs. The study suggested a role of SSRs in the formation of mature RNA isoforms via alternative splicing events during stress response. A similar association of miRNA-SSRs with the gene expressions and phenotypic manifestations has also been reported in rice (Mondal and Ganie, 2014; Ganie and Mondal, 2015).

## Conclusion

Here we reported integrated analysis of sequence data on genome, transcriptome and nc RNAs to generate trait specific novel DNA markers like miRNA-SSRs and EST-SSRs for seed type trait. *In silico* analysis enabled the development of 132 miRNA-SSR primers for seed type trait from 761 pre-miRNA sequences. One hundred twenty three markers were successfully validated and mapped onto Tunisia chromosomes to generate physical maps. Further, identified 63 highly polymorphic markers out of 80 miRNA-SSR primers producing unique amplicons through ePCR validation across multiple genomes of pomegranate. PCR validation and genetic diversity analysis confirmed the utility of these novel markers for future research and breeding. Following target prediction and SSR mining in resulting 1935 unique genes, we developed 413 EST-SSR markers with possible association with seed type trait. Besides, we demonstrated the utility of these markers in identifying master miRNAs and their candidate genes thorough target analysis, functional annotations of target genes and their KEGG pathways and network analysis. We identified a subset of five informative miRNA-SSRs (miRNA_SH_SSR69, miRNA_SH_SSR36, miRNA_SH_SSR103, miRNA_SH_SSR35 and miRNA_SH_SSR53) that influence functioning of several genes involved in seed hardness process. The potential miRNAs and their candidate targets identified here create new avenues for gene editing applications for improving seed type trait in pomegranate.

## Data Availability

The original contributions presented in the study are included in the article/Supplementary Materials, further inquiries can be directed to the corresponding author.

## Supplementary Material

Supplementary Table 1 Details of 16 pomegranate genotypes used in this study.

Supplementary Table 2 Details of 132 miRNA_SH_SSR primers designed in relation to seed hardness trait in pomegranate.

Supplementary Table 3 Details of physical locations of 123 miRNA-SSR markers which are distributed across the 8 chromosomes of Tunisia.

Supplementary Table 4 Marker statistics as computed for 80 miRNA-SSR primers screened through ePCR on four pomegranate genomes.

Supplementary Table 5 Details of 1935 unique gene targets with functional details which are targeted by 24 miRNA families in this study.

Supplementary Table 6 Details of miRNA-SSRs, their families and the target candidate genes engaged in seed development and maturation.

Supplementary Table 7 Details of 58 KEGG pathways for annotated miRNA-target genes with enzymes details.

Supplementary Table 8 Details of top 754 target genes based on lowest alignment score and highest mfe which are targeted by 24 miRNA families.

Supplementary Table 9 Details of 7688 SSR motifs and 413 EST-SSR primers designed based on 1935 unique targets of 24 miRNA families.

Supplementary Table 10 List of 32 pre-miRNA-SSRs initially validated on six pomegranate genotypes.

Supplementary Table 11 Genetic diversity statistics of ten MIR_SH_ SSRs obtained from 16 pomegranate genotypes.

Supplementary Figure 1 EST-SSR marker statistics and frequency distribution graphs of different SSR motifs in 1935 unique target genes of 24 miRNA families.

Supplementary Figure 2 Allelic variations among **(A)** Six pomegranate genotypes using MIR_SH_SSR markers 64, 65, 67, 69, 71, 81, 84 and 86, and **(b)** Sixteen pomegranate genotypes using MIR_SH_SSRs 26, 29 and 37. (Where, L-100 bp DNA ladder, Lane 1–6 and Lane 1–16 set of pomegranate genotypes as mentioned in material and methods and listed in Supplementary Table S1)

## Abbreviations

miRNA, Micro RNA; ePCR, Electronic polymerase chain reaction; PIC, Polymorphism information content; QTL, Quantitative trait loci; UPGMA, Unweighted pair group method of arithmetic averages.
