# Supplementary material for: Novel miRNA-SSRs for Improving Seed Hardness Trait of Pomegranate (Punica granatum L.)
Source: Front Genet. 2022 Apr 12;13:866504. doi: 10.3389/fgene.2022.866504 (PMC9040167; doi:10.3389/fgene.2022.866504)
Supplement: Supplementary file 3 [file Table1.DOCX]

**Suppl. Table 1** Details of 16 pomegranate genotypes used in this study

| **Sl. No** | **Genotype Name** | **Code** | **Seed Type** | **Type** | **Origin/source** |
| --- | --- | --- | --- | --- | --- |
| **1** | Jyoti | JYT | Soft | Commercial variety | India (Karnataka) |
| **2** | G-137 | G137 | Soft | Commercial variety | India (Maharashtra) |
| **3** | Ganesh | GNS | Soft | Commercial Variety | India (Maharashtra) |
| **4** | Arakta | ARKT | Soft | Commercial Variety | India (Maharashtra) |
| **5** | Mridula | MRD | Soft | Commercial variety | India (Maharashtra) |
| **6** | Jallore Seedless | JS | Soft | Cultivar | India (Rajasthan) |
| **7** | Kandhari | KND | Soft | Exotic Breeding Line | Afghanistan |
| **8** | Ruby | RUB | Soft | Commercial Variety | India (Karnataka) |
| **9** | Co-White | CWT | Hard | Cultivar | India (Tamil Nadu) |
| **10** | Kalpitiya | KALP | Hard | Exotic Variety | Sri Lanka |
| **11** | Yercaud | YERC | Hard | Cultivar | India (Tamil Nadu) |
| **12** | Kabuli Yellow | KABY | Hard | Exotic Breeding Line | Afghanistan |
| **13** | Jodhpur Red | JDR | Hard | Cultivar | India (Rajasthan) |
| **14** | Jodhpur Collection | JDC | Soft | Cultivar | India (Rajasthan) |
| **15** | Gulesha Red | GULR | Soft | Exotic Cultivar | Russia |
| **16** | Tabesta | TABS | Hard | Exotic Cultivar | Iran |
