# Supplementary figures and images for: Novel miRNA-SSRs for Improving Seed Hardness Trait of Pomegranate (Punica granatum L.)
Source: Front Genet. 2022 Apr 12;13:866504. doi: 10.3389/fgene.2022.866504 (PMC9040167; doi:10.3389/fgene.2022.866504)

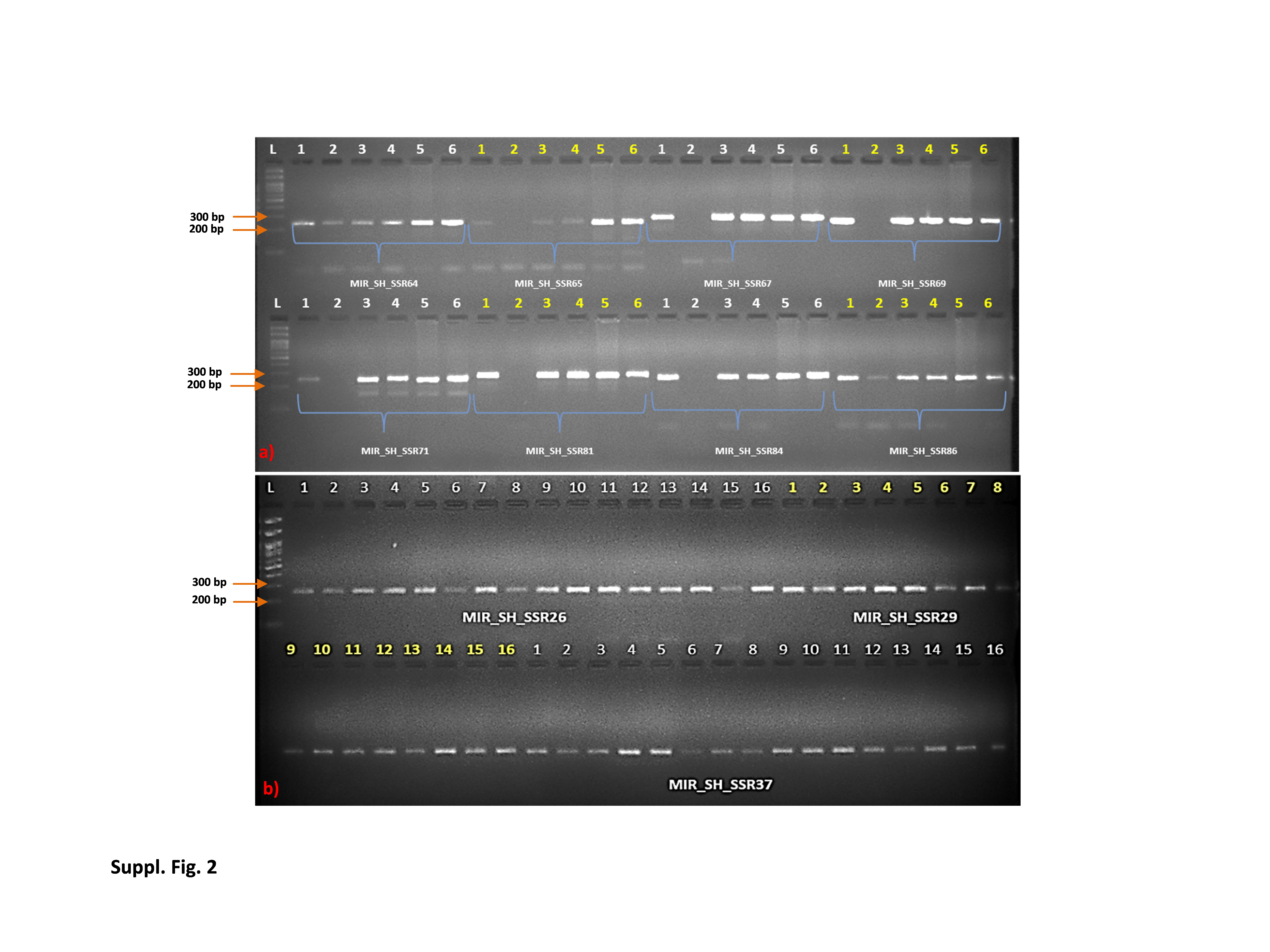

Supplement: Supplementary file 5 [file Image2.TIF]

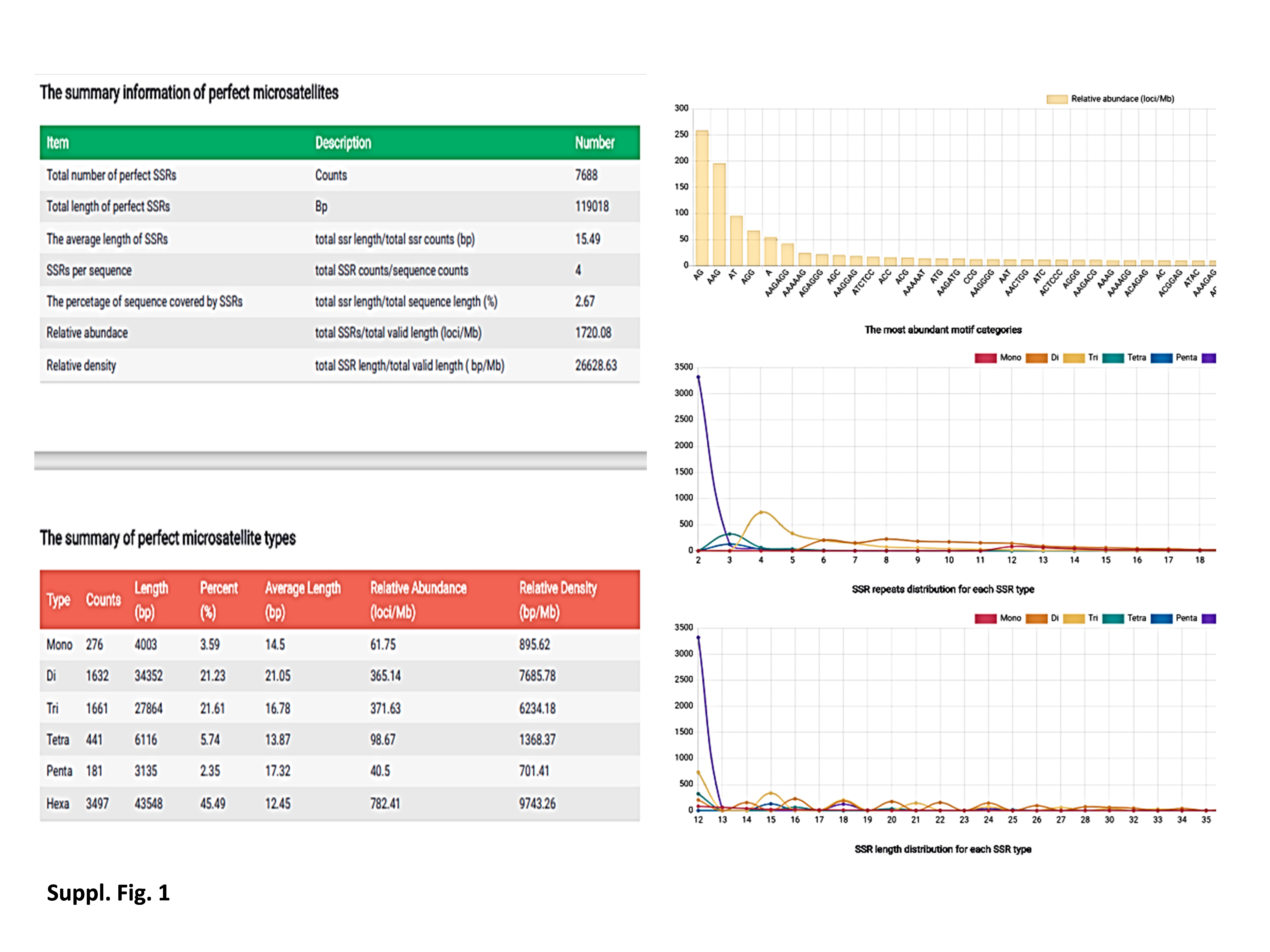

Supplement: Supplementary file 6 [file Image1.TIF]
