# Supplementary material for: Novel miRNA-SSRs for Improving Seed Hardness Trait of Pomegranate (Punica granatum L.)
Source: Front Genet. 2022 Apr 12;13:866504. doi: 10.3389/fgene.2022.866504 (PMC9040167; doi:10.3389/fgene.2022.866504)
Supplement: Supplementary file 7 [file Table4.DOCX]

**Suppl. Table S4.** Marker statistics as computed for 80 miRNA-SSR primers screened through ePCR on four pomegranate genomes

| **SL.No** | **Marker** | **Na** | **MAF** | **Ne** | ***I*** | ***Ho*** | ***He*** | **PIC** |
| --- | --- | --- | --- | --- | --- | --- | --- | --- |
| 1 | miRNA_SSR117 | 2 | 0.75 | 1.60 | 0.56 | 0.50 | 0.38 | 0.43 |
| 2 | miRNA_SSR131 | 3 | 0.50 | 2.46 | 0.97 | 1.00 | 0.59 | 0.68 |
| 3 | miRNA_SSR67 | 3 | 0.50 | 2.46 | 0.97 | 1.00 | 0.59 | 0.68 |
| 4 | miRNA_SSR75 | 4 | 0.50 | 2.91 | 1.21 | 1.00 | 0.66 | 0.75 |
| 5 | miRNA_SSR92 | 2 | 0.50 | 2.00 | 0.69 | 1.00 | 0.50 | 0.57 |
| 6 | miRNA_SSR93 | 3 | 0.50 | 2.57 | 1.01 | 1.00 | 0.61 | 0.73 |
| 7 | miRNA_SSR94 | 2 | 0.63 | 1.88 | 0.66 | 0.75 | 0.47 | 0.54 |
| 8 | miRNA_SSR103 | 3 | 0.50 | 2.46 | 0.97 | 0.75 | 0.59 | 0.68 |
| 9 | miRNA_SSR104 | 2 | 0.50 | 2.00 | 0.69 | 1.00 | 0.50 | 0.57 |
| 10 | miRNA_SSR105 | 2 | 0.50 | 2.00 | 0.69 | 1.00 | 0.50 | 0.57 |
| 11 | miRNA_SSR106 | 2 | 0.75 | 1.60 | 0.56 | 0.50 | 0.38 | 0.43 |
| 12 | miRNA_SSR107 | 2 | 0.75 | 1.60 | 0.56 | 0.50 | 0.38 | 0.43 |
| 13 | miRNA_SSR114 | 2 | 0.50 | 2.00 | 0.69 | 1.00 | 0.50 | 0.57 |
| 14 | miRNA_SSR126 | 4 | 0.50 | 2.91 | 1.21 | 1.00 | 0.66 | 0.75 |
| 15 | miRNA_SSR26 | 2 | 0.63 | 1.88 | 0.66 | 0.75 | 0.47 | 0.54 |
| 16 | miRNA_SSR31 | 3 | 0.63 | 2.13 | 0.90 | 0.75 | 0.53 | 0.61 |
| 17 | miRNA_SSR32 | 3 | 0.50 | 2.67 | 1.04 | 0.50 | 0.63 | 0.71 |
| 18 | miRNA_SSR35 | 3 | 0.75 | 1.68 | 0.74 | 0.50 | 0.41 | 0.46 |
| 19 | miRNA_SSR36 | 4 | 0.50 | 2.91 | 1.21 | 1.00 | 0.66 | 0.75 |
| 20 | miRNA_SSR37 | 2 | 0.75 | 1.60 | 0.56 | 0.50 | 0.38 | 0.43 |
| 21 | miRNA_SSR47 | 2 | 0.50 | 2.00 | 0.69 | 1.00 | 0.50 | 0.57 |
| 22 | miRNA_SSR49 | 4 | 0.63 | 2.29 | 1.07 | 0.50 | 0.56 | 0.64 |
| 23 | miRNA_SSR50 | 3 | 0.50 | 2.67 | 1.04 | 1.00 | 0.63 | 0.71 |
| 24 | miRNA_SSR51 | 4 | 0.50 | 2.91 | 1.21 | 1.00 | 0.66 | 0.75 |
| 25 | miRNA_SSR52 | 3 | 0.50 | 2.67 | 1.04 | 0.50 | 0.63 | 0.71 |
| 26 | miRNA_SSR66 | 3 | 0.38 | 2.91 | 1.08 | 0.75 | 0.66 | 0.75 |
| 27 | miRNA_SSR77 | 2 | 0.50 | 2.00 | 0.69 | 1.00 | 0.50 | 0.57 |
| 28 | miRNA_SSR79 | 3 | 0.63 | 2.13 | 0.90 | 0.50 | 0.53 | 0.61 |
| 29 | miRNA_SSR8 | 3 | 0.50 | 2.67 | 1.04 | 1.00 | 0.63 | 0.71 |
| 30 | miRNA_SSR81 | 4 | 0.38 | 3.20 | 1.26 | 1.00 | 0.69 | 0.79 |
| 31 | miRNA_SSR108 | 2 | 0.50 | 2.00 | 0.69 | 1.00 | 0.50 | 0.57 |
| 32 | miRNA_SSR39 | 2 | 0.50 | 2.00 | 0.69 | 1.00 | 0.50 | 0.57 |
| 33 | miRNA_SSR41 | 3 | 0.67 | 2.00 | 0.87 | 0.33 | 0.50 | 0.60 |
| 34 | miRNA_SSR43 | 3 | 0.63 | 2.13 | 0.90 | 0.75 | 0.53 | 0.61 |
| 35 | miRNA_SSR44 | 3 | 0.50 | 2.67 | 1.04 | 1.00 | 0.63 | 0.71 |
| 36 | miRNA_SSR68 | 2 | 0.75 | 1.60 | 0.56 | 0.50 | 0.38 | 0.43 |
| 37 | miRNA_SSR10 | 1 | 1.00 | 1.00 | 0.00 | 0.00 | 0.00 | 0.00 |
| 38 | miRNA_SSR17 | 3 | 0.50 | 2.67 | 1.04 | 1.00 | 0.63 | 0.71 |
| 39 | miRNA_SSR18 | 3 | 0.50 | 2.57 | 1.01 | 1.00 | 0.61 | 0.73 |
| 40 | miRNA_SSR19 | 2 | 0.88 | 1.28 | 0.38 | 0.25 | 0.22 | 0.25 |
| 41 | miRNA_SSR22 | 3 | 0.50 | 2.46 | 0.97 | 1.00 | 0.59 | 0.68 |
| 42 | miRNA_SSR23 | 2 | 0.63 | 1.88 | 0.66 | 0.75 | 0.47 | 0.54 |
| 43 | miRNA_SSR57 | 2 | 0.50 | 2.00 | 0.69 | 1.00 | 0.50 | 0.57 |
| 44 | miRNA_SSR69 | 4 | 0.50 | 2.91 | 1.21 | 1.00 | 0.66 | 0.75 |
| 45 | miRNA_SSR70 | 2 | 0.50 | 2.00 | 0.69 | 1.00 | 0.50 | 0.57 |
| 46 | miRNA_SSR9 | 2 | 0.50 | 2.00 | 0.69 | 1.00 | 0.50 | 0.57 |
| 47 | miRNA_SSR122 | 2 | 0.50 | 2.00 | 0.69 | 1.00 | 0.50 | 0.60 |
| 48 | miRNA_SSR3 | 3 | 0.63 | 2.13 | 0.90 | 0.75 | 0.53 | 0.61 |
| 49 | miRNA_SSR82 | 2 | 0.75 | 1.60 | 0.56 | 0.50 | 0.38 | 0.43 |
| 50 | miRNA_SSR83 | 3 | 0.50 | 2.67 | 1.04 | 1.00 | 0.63 | 0.71 |
| 51 | miRNA_SSR86 | 3 | 0.50 | 2.67 | 1.04 | 0.50 | 0.63 | 0.71 |
| 52 | miRNA_SSR95 | 2 | 0.63 | 1.88 | 0.66 | 0.75 | 0.47 | 0.54 |
| 53 | miRNA_SSR96 | 3 | 0.50 | 2.46 | 0.97 | 1.00 | 0.59 | 0.68 |
| 54 | miRNA_SSR101 | 2 | 0.63 | 1.88 | 0.66 | 0.75 | 0.47 | 0.54 |
| 55 | miRNA_SSR111 | 2 | 0.50 | 2.00 | 0.69 | 1.00 | 0.50 | 0.57 |
| 56 | miRNA_SSR115 | 1 | 1.00 | 1.00 | 0.00 | 0.00 | 0.00 | 0.00 |
| 57 | miRNA_SSR116 | 2 | 0.50 | 2.00 | 0.69 | 1.00 | 0.50 | 0.57 |
| 58 | miRNA_SSR128 | 2 | 0.50 | 2.00 | 0.69 | 1.00 | 0.50 | 0.57 |
| 59 | miRNA_SSR20 | 3 | 0.50 | 2.46 | 0.97 | 1.00 | 0.59 | 0.68 |
| 60 | miRNA_SSR21 | 3 | 0.75 | 1.68 | 0.74 | 0.25 | 0.41 | 0.46 |
| 61 | miRNA_SSR4 | 3 | 0.50 | 2.46 | 0.97 | 1.00 | 0.59 | 0.68 |
| 62 | miRNA_SSR5 | 3 | 0.75 | 1.68 | 0.74 | 0.25 | 0.41 | 0.46 |
| 63 | miRNA_SSR84 | 1 | 1.00 | 1.00 | 0.00 | 0.00 | 0.00 | 0.00 |
| 64 | miRNA_SSR13 | 3 | 0.63 | 2.13 | 0.90 | 0.75 | 0.53 | 0.61 |
| 65 | miRNA_SSR14 | 3 | 0.50 | 2.46 | 0.97 | 1.00 | 0.59 | 0.68 |
| 66 | miRNA_SSR53 | 3 | 0.75 | 1.68 | 0.74 | 0.25 | 0.41 | 0.46 |
| 67 | miRNA_SSR54 | 3 | 0.50 | 2.46 | 0.97 | 1.00 | 0.59 | 0.68 |
| 68 | miRNA_SSR60 | 2 | 0.63 | 1.88 | 0.66 | 0.75 | 0.47 | 0.54 |
| 69 | miRNA_SSR71 | 4 | 0.63 | 2.29 | 1.07 | 0.75 | 0.56 | 0.64 |
| 70 | miRNA_SSR72 | 3 | 0.75 | 1.68 | 0.74 | 0.25 | 0.41 | 0.46 |
| 71 | miRNA_SSR73 | 2 | 0.50 | 2.00 | 0.69 | 1.00 | 0.50 | 0.60 |
| 72 | miRNA_SSR74 | 2 | 0.50 | 2.00 | 0.69 | 1.00 | 0.50 | 0.57 |
| 73 | miRNA_SSR97 | 3 | 0.50 | 2.46 | 0.97 | 1.00 | 0.59 | 0.68 |
| 74 | miRNA_SSR98 | 2 | 0.88 | 1.28 | 0.38 | 0.25 | 0.22 | 0.25 |
| 75 | miRNA_SSR1 | 3 | 0.50 | 2.67 | 1.04 | 1.00 | 0.63 | 0.71 |
| 76 | miRNA_SSR120 | 4 | 0.38 | 3.56 | 1.32 | 1.00 | 0.72 | 0.82 |
| 77 | miRNA_SSR121 | 2 | 0.63 | 1.88 | 0.66 | 0.75 | 0.47 | 0.54 |
| 78 | miRNA_SSR34 | 6 | 0.25 | 5.33 | 1.73 | 1.00 | 0.81 | 0.93 |
| 79 | miRNA_SSR88 | 2 | 0.63 | 1.88 | 0.66 | 0.75 | 0.47 | 0.54 |
| 80 | miRNA_SSR89 | 3 | 0.75 | 1.68 | 0.74 | 0.50 | 0.41 | 0.46 |
|  |  | **2.66** | **0.58** | **2.19** | **0.82** | **0.77** | **0.51** | **0.58** |

Note* Na: Numbers of alleles; MAF: Major Allelic Frequency; Ne: Number of Effective Alleles; *I*: Shannon’s Information Index; *Ho*: Observed heterozygosity; *He*: Expected heterozygosity; PIC: Polymorphic Information Content
