# Supplementary material for: Novel miRNA-SSRs for Improving Seed Hardness Trait of Pomegranate (Punica granatum L.)
Source: Front Genet. 2022 Apr 12;13:866504. doi: 10.3389/fgene.2022.866504 (PMC9040167; doi:10.3389/fgene.2022.866504)
Supplement: Supplementary file 9 [file Table11.DOCX]

**Suppl. Table S11.** Genetic diversity statistics of 10 MIR_SH_ SSRs obtained from 16 pomegranate genotypes

| **SL.No** | **Primer Seq ID** | **Chromosome**  **location** | **Na** | **Ne** | **MAF** | ***He*** | ***I*** | **PIC** |
| --- | --- | --- | --- | --- | --- | --- | --- | --- |
| **1** | **MIR_SH_SSR11** | 4 | 2 | 1.74 | 0.69 | 0.43 | 0.62 | 0.44 |
| **2** | **MIR_SH_SSR13** | 7 | 2 | 1.44 | 0.81 | 0.30 | 0.48 | 0.32 |
| **3** | **MIR_SH_SSR23** | 4 | 2 | 1.97 | 0.56 | 0.49 | 0.69 | 0.51 |
| **4** | **MIR_SH_SSR25** | 4 | 2 | 1.47 | 0.80 | 0.32 | 0.50 | 0.33 |
| **5** | **MIR_SH_SSR26** | 2 | 2 | 2.00 | 0.50 | 0.50 | 0.69 | 0.52 |
| **6** | **MIR_SH_SSR29** | - | 2 | 1.44 | 0.81 | 0.30 | 0.48 | 0.32 |
| **7** | **MIR_SH_SSR37** | 2 | 2 | 2.00 | 0.50 | 0.50 | 0.69 | 0.52 |
| **8** | **MIR_SH_SSR64** | 2 | 2 | 1.85 | 0.64 | 0.46 | 0.65 | 0.48 |
| **9** | **MIR_SH_SSR71** | 7 | 2 | 1.47 | 0.80 | 0.32 | 0.50 | 0.33 |
| **10** | **MIR_SH_SSR86** | 5 | 2 | 1.30 | 0.87 | 0.23 | 0.39 | 0.24 |
|  |  | **Mean** | **2(22)** | **1.67** | **0.70** | **0.39** | **0.57** | **0.40** |

Note* Na: Numbers of alleles; Ne: Number of Effective Alleles; MAF: Major Allelic Frequency; *He*: Expected heterozygosity; *I*: Shannon’s Information Index; PIC: Polymorphic Information Content.
