# Supplementary material for: Novel miRNA-SSRs for Improving Seed Hardness Trait of Pomegranate (Punica granatum L.)
Source: Front Genet. 2022 Apr 12;13:866504. doi: 10.3389/fgene.2022.866504 (PMC9040167; doi:10.3389/fgene.2022.866504)
Supplement: Supplementary file 10 [file Table6.DOCX]

**Suppl. Table S6.** Details of miRNA-SSRs, their families and the target candidates genes engaged in seed development and maturation

| **Sl.No** | **miRNA-SSR** | **Gene ID** | **miRNA** | **Family** | **Predicted gene targets of miRNAs** |
| --- | --- | --- | --- | --- | --- |
| **1** | **MIR_SH_SSR69** | >gi\|1208532759\|gb\|MTKT01001287.1\|:3914139-3915299_535_654_20 cand1 | ath-MIR156b | UCUCUCUCUCUCUCUCUCUCUC | transcription factor MYB1-like (LOC116187398),auxin response factor 17 (LOC116188553),probable pectate lyase 8 (LOC116189201),cellulose synthase-like protein E6 (LOC116189990),auxin response factor 9-like (LOC116192073), cellulose synthase A catalytic subunit 3 [UDP-forming]-like (LOC116192084),glucan endo-1,3-beta-glucosidase 13-like (LOC116200898),glucan endo-1,3-beta-glucosidase 11 (LOC116200925),AP2/ERF and B3 domain-containing transcription factor At1g50680-like (LOC116201010), ABC transporter C family member 13 (LOC116202087), WRKY transcription factor 22 (LOC116202559), NAC domain-containing protein 10 (LOC116206330), |
| **2** | **MIR_SH_SSR51** | >gi\|1208524004\|gb\|MTKT01002492.1\|:3833598-3835006_618_735_20 cand3 | ath-MIR157c | UCUCUCUCUCAUCUCGAACCAG  AUUCGAGAAAGAGAGAGAGAGA | GTP-binding protein SAR1A-like (LOC116188377),septum site-determining protein minD homolog, chloroplastic (LOC116203451),protein EXPORTIN 1A-like (LOC116204994),phosphoinositide phosphatase SAC3 (LOC116188417) |
| **3** | **MIR_SH_SSR39** | >gi\|1208504359\|gb\|MTKT01005556.1\|:2733576-2736135_1987_2090_20 cand3 | ath-MIR167d | UCGGUCGGCAAAUCCUUAUCAU | zinc metalloprotease EGY2, chloroplastic (LOC116189522) |
| **4** | **MIR_SH_SSR 16** | >gi\|1208537849\|gb\|MTKT01000790.1\|:174503-176495_1103_1209_20 cand6 | ath-MIR169c_1 | UGGCAUAGAUGCUUAAUGUGAU | kinesin-like protein NACK2 (LOC116188415) |
| **5** | **MIR_SH_SSR26** | >gi\|1208529806\|gb\|MTKT01001935.1\|:698201-699199_482_593_20 cand1 | ath-MIR169c_2 | AAGGUAUAUUUUAUCGUGCUUG | protein transport protein SEC16A homolog (LOC116192378) |
| **6** | **MIR_SH_SSR15** | >gi\|1208537849\|gb\|MTKT01000790.1\|:174503-176495_1740_1840_20 cand7 | ath-MIR395f | UCAUGUAUUGUGAUAAAUUGGG | pentatricopeptide repeat-containing protein At1g56570 (LOC116208018) |
| **7** | **MIR_SH_SSR37** | >gi\|1208510000\|gb\|MTKT01004810.1\|:4681114-4683510_571_673_20 cand4 | ath-MIR401 | CUAAAUUAAUACUAGACUUUUU | pentatricopeptide repeat-containing protein At5g08490 (LOC116202200) |
| **8** | **MIR_SH_SSR31** | >gi\|1208512650\|gb\|MTKT01004399.1\|:2907713-2909392_919_1015_20 cand2 | ath-MIR406_1 | GCUAGCUAGCUAGGUUUUUUUU | xyloglucan endotransglucosylase/hydrolase protein 23 (LOC116200953) |
| **9** | **MIR_SH_SSR90** | >gi\|1208540229\|gb\|MTKT01000548.1\|:1463712-1464886_499_602_20 cand1 | ath-MIR406_2 | CCGUUUGGAUUCAGAGUUAAAG  GAUUUUGAUUGUGGAAAAGGAC | uncharacterized LOC116204960 (LOC116204960),extensin-like (LOC116203544) |
| **10** | **MIR_SH_SSR18** | >gi\|1208506790\|gb\|MTKT01005376.1\|:626200-627211_478_593_20 cand4 | ath-MIR407 | CGUUUGGAUUCAGAGUUAAAGU  AUUGUGAAAAAGGACAAAUGAG | uncharacterized LOC116204960 (LOC116204960),extensin-like (LOC116203544) |
| **11** | **MIR_SH_SSR54** | >gi\|1208501614\|gb\|MTKT01005880.1\|:1149767-1151372_216_315_20 cand2 | ghr-MIR482b | UGGUGACCUCGGUGGAGGGGUC | DNA-directed RNA polymerase III subunit RPC6 (LOC116187952) |
| **12** | **MIR_SH_SSR86** | >gi\|1208506003\|gb\|MTKT01005400.1\|:2941708-2943282_542_645_20 cand1 | ptr-mir-564 | CGGUGUAUGAAGGAACGAGAAC | small heat shock protein, chloroplastic (LOC116203919) |
| **13** | **MIR_SH_SSR130** | >gi\|1208526698\|gb\|MTKT01002214.1\|:9131484-9132557_499_599_20 cand2 | ath-MIR836 | AUUGGAGCCCAUUUGCAACUCA  AGUUGCAGAUGGGCUCUAGUCC | uncharacterized LOC116200611 (LOC116200611) |
| **14** | **MIR_SH_SSR14** | >gi\|1208537849\|gb\|MTKT01000790.1\|:1321801-1322799_85_187_20 cand1 | ath-MIR838 | UGUCAGGAUCUGGAGUCGGCUC | uncharacterized LOC116187690 (LOC116187690) |
| **15** | **MIR_SH_SSR64** | >gi\|1208506917\|gb\|MTKT01005370.1\|:300790-301803_432_526_20 cand1 | ath-MIR857 | AUAUGGAAACAGUAAGUGAGAC | protein DETOXIFICATION 14-like (LOC116204529) |
| **16** | **MIR_SH_SSR23** | >gi\|1208502974\|gb\|MTKT01005739.1\|:1575408-1576802_650_760_20 cand2 | ath-MIR859 | AGUUUGGAUUUUCUUCAUUCAU | serine/threonine-protein kinase D6PKL2-like (LOC116204987),NDR1/HIN1-like protein 6 (LOC116215187) |
| **17** | **MIR_SH_SSR71** | >gi\|1208511334\|gb\|MTKT01004609.1\|:2735763-2737255_419_523_20 cand1 | ath-MIR863 | UAUAUAUAUAUUAAAGAAGCAC | uncharacterized LOC116207084 (LOC116207084) |
| **18** | **MIR_SH_SSR12** | >gi\|1208537849\|gb\|MTKT01000790.1\|:1321801-1322799_459_579_20 cand4 | ath-MIR5021_1 | UCUCUUCUUCUUCUUCUUCGGG  AGAAUUGAAGAAGAAAAGAAGA | uncharacterized LOC116208259 (LOC116208259),LRP chaperone MESD (LOC116205388) |
| **19** | **MIR_SH_ SSR36** | >gi\|1208510000\|gb\|MTKT01004810.1\|:4681114-4683510_1168_1285_20 cand8 | ath-MIR5021_2 | UUUCUUCUUCUUCUUCGAGAAC  ACGAGAAGAAGAAGAAGAAGAA | uncharacterized vacuolar membrane protein YML018C (LOC116187164),receptor protein-tyrosine kinase CEPR2 (LOC116203618),myosin heavy chain, clone 203 (LOC116215093),resistance RPP13-like protein 4 (LOC116188985),fasciclin-like arabinogalactan protein 19 (LOC116203232),pentatricopeptide repeat-containing protein At3g63370, chloroplastic (LOC116215830),alpha/beta hydrolase domain-containing protein 17B (LOC116204711) |
| **20** | **MIR_SH_SSR29** | >gi\|1208512650\|gb\|MTKT01004399.1\|:919800-921144_1132_1250_20 cand3 | ath-MIR5631 | UUUCUUUUUCUUAAAUAAGAUU | 50S ribosomal protein L21, mitochondrial (LOC116206400), |
| **21** | **MIR_SH_SSR 41** | >gi\|1208504359\|gb\|MTKT01005556.1\|:2733576-2736135_314_417_20 cand1 | ath-MIR5638a | GAUGGAAAAAUCUAGUAAAGAA  CAUUUAUUUAUUUUUUGGUUGG | phosphatidate phosphatase PAH2-like (LOC116191056) |
| **22** | **MIR_SH_SSR 11** | >gi\|1208525649\|gb\|MTKT01002371.1\|:24603-25603_703_805_20 cand2 | ath-MIR5640 | CACCAUGUUAUAUUUCUUGGGU  CCCAUAUUUAUUACUAAUACUA | (R,S)-reticuline 7-O-methyltransferase-like (LOC116205650) |
| **23** | **MIR_SH_SSR84** | >gi\|1208519617\|gb\|MTKT01003224.1\|:3780474-3781739_603_720_20 cand2 | ath-MIR5645f | GAAAAAUGACAAAUGAGAUAUG  GUUGUGUAGUGUGUUGAGUUAA | serine/threonine-protein kinase AFC3 (LOC116213661) |
| **24** | **MIR_SH_SSR3** | >gi\|1208526095\|gb\|MTKT01002270.1\|:542601-543604_560_663_20 cand2 | ath-MIR5651_1 | AUGCAAAAUAUAUAUAUAUAUA  AUAUAUAUAUUUCGAUGCAUAA | F-box/kelch-repeat protein At1g55270-like (LOC116201018) |
| **25** | **MIR_SH_SSR 48** | >gi\|1208526286\|gb\|MTKT01002229.1\|:1113193-1114190_829_933_20 cand4 | ath-MIR5651_2 | AUAUAUACCUACUACACAAGUU | regulator of nonsense transcripts 1 homolog (LOC116203386) |
| **26** | **MIR_SH_SSR103** | >gi\|1208514145\|gb\|MTKT01004273.1\|:740053-741440_717_815_20 cand3 | ath-MIR5651_3 | UUAUAUGUAUAUAUUUUGAUAA  AUAUAUAUAUAUAUAUAUAUUA | UDP-glucose 6-dehydrogenase 3-like (LOC116187099),lignin-forming anionic peroxidase-like (LOC116202760), cellulose synthase A catalytic subunit 3 [UDP-forming] (LOC116187640), |
| **27** | **MIR_SH_SSR35** | >gi\|1208510000\|gb\|MTKT01004810.1\|:4681114-4683510_1121_1250_20 cand6 | ath-MIR5655 | CUUCUUCUUCUUCUUCUUCUUC  ACAGGGUGAGGGAAGGGAGGAG | probable pectate lyase 8 (LOC116189201),UDP-N-acetylglucosamine transporter UGNT1 (LOC116189358),AP2/ERF and B3 domain-containing transcription factor At1g50680-like (LOC116201010), polygalacturonate 4-alpha-galacturonosyltransferase (LOC116203706),probable pectinesterase 68 (LOC116206137),WRKY transcription factor 23-like (LOC116215372), BRASSINOSTEROID INSENSITIVE 1-associated receptor kinase 1-like (LOC116205132), auxin response factor 19-like (LOC116187658), myb-like protein X (LOC116204011) |
| **28** | **MIR_SH_SSR24** | >gi\|1208502974\|gb\|MTKT01005739.1\|:2972970-2973879_325_435_20 cand2 | ath-MIR5665 | UUGUGGAAAAUGACAAAUGAGA  CGUGUGUUUUUAUUGUAUAGUG | 60S acidic ribosomal protein P1-like (LOC116192471) |
| **29** | **MIR_SH_SSR53** | >gi\|1208501614\|gb\|MTKT01005880.1\|:1149767-1151372_57_154_20 cand1 | mtr-MIR5253 | UUGGCGACCUCGAUGGAGGGGU | embryogenesis-associated protein EMB8 (LOC116205178) |
| **30** | **MIR_SH_SSR112** | >gi\|1208517326\|gb\|MTKT01003711.1\|:975008-977463_2031_2132_20 cand13 | ame-mir-6042 | GGUGACCUCGGUAGAAGGGUCA | DNA-directed RNA polymerase III subunit RPC6 (LOC116187952) |
| **31** | **MIR_SH_SSR118** | >gi\|1208536493\|gb\|MTKT01000813.1\|:1477993-1479384_476_576_20 cand1 | ath-MIR8180 | CUUACUCUACAAAUAUAUAUAU | resistance protein RPM1-like (LOC116214292) |
