# Supplementary material for: Novel miRNA-SSRs for Improving Seed Hardness Trait of Pomegranate (Punica granatum L.)
Source: Front Genet. 2022 Apr 12;13:866504. doi: 10.3389/fgene.2022.866504 (PMC9040167; doi:10.3389/fgene.2022.866504)
Supplement: Supplementary file 13 [file Table10.DOCX]

**Suppl. Table S10.** List of pre-32 miRNA-SSRs initially validated on six pomegranate genotypes

| **Sl.No** | **Primer Seq ID** | **Sequence F/R (5’-3’)** | **Chromosome** | **Type of repeat** | **Size range** | **Number of Alleles** | **Polymorphic information content(PIC)** |
| --- | --- | --- | --- | --- | --- | --- | --- |
| **1** | **MIR_SH_SSR11** | TTCTTGGGTCCTTATACCG/CTTCACCTAACAAGCGATG | 4 | (TACTAA)2 | 250-256 | 2 | 0.36 |
| **2** | **MIR_SH_SSR12** | CACTGGTGGTTCAAAAAGTT/TTCTTCTTCTCGTCCTGCT | - | (CT)7 | 245-255 | 2 | 0.54 |
| **3** | **MIR_SH_SSR13** | AGCAGGACGAGAAGAAGAA/GACTTGGGCCAACATTTTA | 7 | (AT)6 | 250-255 | 2 | 0.36 |
| **4** | **MIR_SH_SSR14** | AGGTTGTCAGGATCTGGAG/TGTACCATCGAGTAGCAAAA | 7 | (TCGATC)2 | 255-260 | 2 | 0.48 |
| **5** | **MIR_SH_SSR15** | GGACAATGTCAGTGCAGTC/ATGCCATCATCATCACATC | 7 | (TATC)4 | 256-260 | 2 | 0.30 |
| **6** | **MIR_SH_SSR16** | CCCATCCTTTCTCCTATCA/AGGGTTAGTGACCCATTTG | 7 | (AAAATT)3 | 250-255 | 2 | 0.48 |
| **7** | **MIR_SH_SSR18** | TTCGAGAGAGTACTCGGTGT/CACACATTTTCCAGGTCAA | 4 | (ATTTTG)5 | 244-250 | 2 | 0.30 |
| **8** | **MIR_SH_SSR23** | ATTTGGAGAATTGGTGAGG/TGGTTTAAGCAGTTTGGTG | 4 | (ATTCAA)2 | 254-260 | 2 | 0.48 |
| **9** | **MIR_SH_SSR24** | TGAGCGTTGATATATTGTGC/AAACACACGTTTCCCAAGT | 4 | (ATTTTG)3 | - | - | na |
| **10** | **MIR_SH_SSR25** | CCTCATTCTGACACCAAAA/GGTTCCACCTGATGATGTA | 4 | (AGTTAA)4 | 255-260 | 2 | 0.48 |
| **11** | **MIR_SH_SSR26** | GTGCCGTTTTATCAATTCC/CGATGCGAATCCTAATTCT | 2 | (ATCATG)2 | 250-255 | 2 | 0.48 |
| **12** | **MIR_SH_SSR28** | TCAAACATGCAACATCTCG/CGTCATATATTGTGCTGGAA | - | (AATTAA)2 | 245-250 | 2 | 0.48 |
| **13** | **MIR_SH_SSR29** | AGGGTGCAAAAGTGAGATT/ACGTCCATCGTCTTGGTAT | - | (TTTCTT)2 | 246-250 | 2 | 0.30 |
| **14** | **MIR_SH_SSR31** | CGGATATCAAAATGCACAC/TCGACTTTCTTTTCCCACT | 2 | (TAGC)3 | 255-260 | 2 | 0.54 |
| **15** | **MIR_SH_SSR35** | GCCGAAACAGTAACCTCTC/CCCCCTCATCTTTCTTCTT | 2 | (TTC)15 | - | - | na |
| **16** | **MIR_SH_SSR37** | TTAACGGAATGTTGTGTGG/AACCATTCGTACCCCTTTA | 2 | (GAAATC)2 | 240-245 | 2 | 0.36 |
| **17** | **MIR_SH_SSR41** | GGTGATGATGGAATTACCTA/GTATGCAATGCTCAATTGGT | 3 | (ATTT)3 | 300 | 1 | 0.00 |
| **18** | **MIR_SH_SSR47** | CGGCCTACTAAGGGCTAT/GACGTGGCAAAGTAAGAGA | 2 | (TA)8 | 260-265 | 2 | 0.30 |
| **19** | **MIR_SH_SSR48** | AAAGAGAGGCAGTCTTTGC/AGTCCGCAAATACACCAA | 2 | (TA)10 | 115-121 | 2 | 0.53 |
| **20** | **MIR_SH_SSR51** | CCCCTCCCTATCTCTCTCT/TCCCATTATGGTGACTGTG | 2 | (AG)14 | - | - | na |
| **21** | **MIR_SH_SSR53** | AGAAAATCGAAAGGGAAGG/ACCAGTACCCACAGAGGAC | 7 | (GGGGTC)2 | - | - | na |
| **22** | **MIR_SH_SSR55** | GGGTTAGAGCTACCAATCG/TACTCCCCAATCTTTAGCC | 7 | (TGACCC)2 | 260-268 | 2 | 0.53 |
| **23** | **MIR_SH_SSR64** | GCAGCCTTAGAATTTCGAT/ACTCAAATGAATCCTGCTGT | 2 | (AT)6 | 235-240 | 2 | 0.30 |
| **24** | **MIR_SH_SSR65** | TGGGATAAGGTTTCTTGTCA/ACTCTTCCTGTGACCCCTA | 2 | (TA)13 | 225-233 | 2 | 0.53 |
| **25** | **MIR_SH_SSR67** | TTGTGTGTTACCTCGTAGC/GGAAACATCACAAGCCTCT | 1 | (AATAGA)2 | 265-270 | 2 | 0.53 |
| **26** | **MIR_SH_SSR69** | CTGTCACTGCACTCCTCAC/GCAGCTAATCGGCTATACA | 4 | (CT)16 | 245 | 1 | 0.00 |
| **27** | **MIR_SH_SSR71** | ATCCCATTCCTCCCATTA/GGCAACGAGAGAGAGTTTC | 7 | (TA)16 | 244-250 | 2 | 0.53 |
| **28** | **MIR_SH_SSR81** | CATAAGGAGGGGTGAGAGA/GCATCTTGAGGTCATCTAGC | 2 | (TA)10 | 265-270 | 2 | 0.36 |
| **29** | **MIR_SH_SSR84** | TCGTTTCACATACCGAAGA/AGGGTTGAGTGAGATGAGG | 6 | (TTTGAT)6 | 250-257 | 2 | 0.36 |
| **30** | **MIR_SH_SSR86** | GGCAAAAGGTCAAGTAAGC/GCGACGAGTCTCATAGGTT | 5 | (GAACGA)2 | 238-245 | 2 | 0.30 |
| **31** | **MIR_SH_SSR90** | TGAGTACTCGTCGCAATTC/ACACACGTTTTCCAAGTCA | 8 | (ATTTTG)2 | 263 | 1 | 0.00 |
| **32** | **MIR_SH_SSR103** | GAGTCGAGACATTCGTCAA/GGATTGGGCATGAACTTAG | 2 | (TA)10 | 245-250 | 2 | 0.30 |
|  |  |  |  |  | **Mean** | **1.89 (53)** | **0.38** |

Note* na-Not amplified
